# Genome-wide characterization and expression analysis of MADS-box transcription factor gene family in *Perilla frutescens*

**DOI:** 10.3389/fpls.2023.1299902

**Published:** 2024-01-08

**Authors:** Mengjing Liang, Zhongyang Du, Ze Yang, Tao Luo, Chunli Ji, Hongli Cui, Runzhi Li

**Affiliations:** ^1^Institute of Molecular Agriculture and Bioenergy, College of Agriculture, Shanxi Agricultural University, Jinzhong, Shanxi, China; ^2^Key Laboratory of Coastal Biology and Biological Resource Utilization, Yantai Institute of Coastal Zone Research, Chinese Academy of Sciences, Yantai, Shandong, China

**Keywords:** *Perilla frutescens*, MADS transcription factors, genome-wide characterization, expression profiles, lipid metabolism, stress responses

## Abstract

MADS-box transcription factors are widely involved in the regulation of plant growth, developmental processes, and response to abiotic stresses. *Perilla frutescens*, a versatile plant, is not only used for food and medicine but also serves as an economical oil crop. However, the MADS-box transcription factor family in *P. frutescens* is still largely unexplored. In this study, a total of 93 *PfMADS* genes were identified in *P. frutescens* genome. These genes, including 37 Type I and 56 Type II members, were randomly distributed across 20 chromosomes and 2 scaffold regions. Type II PfMADS proteins were found to contain a greater number of motifs, indicating more complex structures and diverse functions. Expression analysis revealed that most *PfMADS* genes (more than 76 members) exhibited widely expression model in almost all tissues. The further analysis indicated that there was strong correlation between some MIKC^C^-type *PfMADS* genes and key genes involved in lipid synthesis and flavonoid metabolism, which implied that these *PfMADS* genes might play important regulatory role in the above two pathways. It was further verified that *PfMADS47* can effectively mediate the regulation of lipid synthesis in *Chlamydomonas reinhardtii* transformants. Using cis-acting element analysis and qRT-PCR technology, the potential functions of six MIKC^C^-type *PfMADS* genes in response to abiotic stresses, especially cold and drought, were studied. Altogether, this study is the first genome-wide analysis of *PfMADS*. This result further supports functional and evolutionary studies of *PfMADS* gene family and serves as a benchmark for related *P. frutescens* breeding studies.

## Introduction

The MADS-box genes are an important class of transcriptional regulators in plants, named after the acronyms of the first four proteins identified (MCM1 in *Saccharomyces cerevisiae*, AGAMOUS in *Arabidopsis thaliana*, DEFICIENS in *goldenseal*, and SRF in human serum response factor) (Shore and Sharrocks, 1995; Lawton-Rauh et al., 2000). The MADS structural domains are located within the four encoded proteins. These domains are highly conserved and are composed of 56-60 amino acids at the N-terminus. They bind to a specific DNA cis-element known as Carg-box and play a role in regulating the spatiotemporal expression of genes (Messenguy and Dubois, 2003). According to multiple studies, MADS-box genes in plants can be categorized into two types: Type I (M type) and Type II (MIKC type) (Becker and Theissen, 2003). The main difference between the two types is that the MIKC type contains three unique characteristic domains in addition to the DNA-binding M domain. These domains are the intermediate (I) domain, the keratin-like (K) domain, and the C-terminal (C) domain, and they are arranged in this specific order (Yang et al., 2003; Kaufmann et al., 2005). This factor is crucial in enabling MADS-box proteins to form dimers or tetramers and carry out their functions (Immink et al., 2009).

Type I can be further classified into Mα, Mβ, and Mγ subtypes based on the phylogenetic relationship, gene structure, and protein structure of the conserved MADS-box domain. On the other hand, type II is divided into MIKC^C^ and MIKC* subtypes, which is determined by the number of exons encoding the I domain and the structure of the K domain (Gramzow and Theissen, 2010). Numerous MADS-box genes have been identified in angiosperms, and their phylogeny has been reconstructed to date. They found that the MIKC^C^-type MADS-box genes can be categorized into 12 subfamilies, namely *AG*, *AGL2/SEP*, *AGL6*, *AGL12*, *AGL15*, *AGL17*, *FLC*, *GGM13*, *STMADS11*, *AP1/SQUA*, *AP3/PI*, and *TM3* (Becker and Theissen, 2003). The evolutionary process of these genes involved numerous duplication events, leading to a vast multigene family (Irish, 2003).

MADS-box genes are prevalent in eukaryotes, spanning across the plant kingdom (Schilling et al., 2020). Extensive research has been conducted on a substantial number of MADS-box genes in both monocotyledonous plants like corn, wheat, millet, and sorghum, and dicotyledonous plants such as tomato, rape, tobacco, and petunia (Arora et al., 2007; Schilling et al., 2018; Wang et al., 2019). The function of this gene family encompasses various stages of plant development, such as seed germination, vegetative growth, floral organ formation, seed coat development, and embryo morphogenesis (Immink et al., 2009). It plays a pivotal role in the overall plant development process. The ABC model of flower organ development is one of the most well-known models, which was later supplemented by the ABCDE model (Coen and Meyerowitz, 1991; Agrawal et al., 2005). According to reports, only a few type I genes have been explored for their biological functions in plants, mainly involved in female gametophyte, embryo, and seed development (Bemer et al., 2008; Kang et al., 2008; Steffen et al., 2008; Smaczniak et al., 2012). For example, AGL23 regulates the development of *Arabidopsis* female gametophytes and is involved in controlling formation of organelles during embryo development (Colombo et al., 2008). Compared to the M and MIKC types, the MIKC*-type has more conserved functions and has been extensively studied (Pelaz et al., 2000; Swiezewski et al., 2009; Molesini et al., 2020). Recent research has demonstrated that MIKC*-type MADS-box genes have a dual function in plant biology. Apart from their involvement in flower organ development and other processes, these genes also play a vital role in regulating substance metabolism in plants. For instance, *CsMADS6* (*AGAMOUS*-like subfamily) in citrus, *MdMADS6* from apple, and *CaMADS-RIN* (*SEP* subfamily) in pepper have been found to have significant regulatory roles in the carotenoid synthesis pathway (Dong et al., 2014; Lu et al., 2018; Li et al., 2022). It has been reported that *PfMADS* genes may be involved in the flavonoid synthesis pathway of *P. frutescens* (Jiang et al., 2020). An *AGAMOUS*-like MADS-box transcription factor has been identified as being involved in the regulation of the lipid anabolism pathway in oil palm (Li et al., 2020; Zhang et al., 2022). Moreover, its regulatory role in abiotic stresses (cold, drought, salt, and other stresses) has been verified in rice, cereals, cotton seeds, *Arabidopsis thaliana*, and other plants (Wu et al., 2021; Zhao et al., 2021; Zhao et al., 2021; Xue et al., 2022). This demonstrates the significant impact of the MADS-box transcription factor family on plants and its involvement in a wide range of regulatory processes.

*P. frutescens* (*Perilla frutescens* (L.) Britt.), an annual herb of the genus *Perilla* L in the Lamiaceae Martinov family (Nitta et al., 2005). It has a rich cultivation history of over 2,000 years and is currently grown in China, Japan, and South Korea, among other Asian countries (Park et al., 2008). *P. frutescens* seeds have a high oil content, with an oil yield ranging from 46% to 58%. Notably, the α-linolenic acid content constitutes more than 65% of the total oil (Zhou et al., 2022). Alpha-linolenic acid is an essential fatty acid for the human body, exhibiting a range of biological activities such as anti-Alzheimer’s disease, anti-viral, anti-bacterial, and anti-allergic properties (Clemente and Cahoon, 2009). These activities offer significant benefits for human health (Luo et al., 2021). In addition, *P. frutescens* leaves come in various colors such as green and purple. The purple leaves are rich in anthocyanin content and are used as a food pigment (Yoshida et al., 1990; Fujiwara et al., 2018; Li et al, 2022). On the other hand, the green leaves of *P. frutescens* contain a high content of bioactive compounds and are often utilized in various applications such as food, skin creams, and medications for atopic dermatitis (Komatsu et al., 2016; Edge et al., 1997). In summary, *P. frutescens* is a versatile plant with numerous benefits. Due to its unique nutrients and active substances, it is not only consumed as food and used in pharmaceuticals, healthcare, and cosmetics, but also holds economic value as an extensively developed and utilized oil crop (Igarashi and Miyazaki, 2013).

The MADS-box transcription factor family has been extensively studied in a variety of plants, but its presence and characteristics in *P. frutescens* have not yet been investigated. In this study, a total of 93 *PfMADS* gene members were identified using bioinformatics analysis methods at the genome-wide level (Zhang et al., 2021). Their chromosomal locations, phylogenetic evolution, conserved motifs, exon-intron structures, and gene expression profiles were investigated. Additionally, cis-acting elements were analyzed to predict their possible functions in *P. frutescens* and their potential roles in abiotic stress responses. These results provide basic information for a better study of the *PfMADS* genes, and lay the foundation for functional studies and the selection of new varieties of *P. frutescens*.

## Methods

### Identification of *PfMADS* transcription factor family members

The genomic data of *P. frutescens* (https://www.ncbi.nlm.nih.gov/genome/?Term=Perilla+rutescens) were downloaded from NCBI GenBank (Zhang et al., 2021) to create a local database. The 108 AtMADS protein sequences were downloaded from the *Arabidopsis* database TAIR (http://www.arabidopsis.org/). To perform a multiple sequence alignment search in the *P. frutescens* local database, the TBtools software (An Integrative Toolkit Developed for Interactive Analyses of Big Biological Data) was utilized with the protein sequence of the AtMADS as an index. Subsequently, the keywords “MADS” and “SRF” were entered into the database to perform a supplementary search. The HMMER-3.1b2 software package (http://hmmer.janelia.org/) (Finn et al., 2011) was utilized to create a Markov model for the Pfam database (Finn et al., 2006). The domain information of the MADS-box genes (PF00319; http://pfam.sanger.ac.uk) was downloaded for protein sequence search. The resulting model was then used to identify potential MADS-box proteins from the *P. frutescens* protein database. Each sequence was manually checked to ensure it met the threshold of an E-value ≤ 1e^-10^. The conserved structural domains of PfMADS were verified using CDD (https://www.ncbi.nlm.nih.gov/Structure/cdd/wrpsb.cgi) and SMART database (http://smart.embl-heidelberg.de/). Repetitive sequences and structurally incomplete sequences were removed, and members of the *PfMADS* gene family were identified. The MADS domains of PfMADS and the AtMADS proteins were clustered using ClustalX2.0 to perform preliminary classification of PfMADS candidates. The basic physicochemical properties such as relative molecular mass and theoretical isoelectric point (pI), of PfMADS proteins were predicted by the online software ProtParam (http://web.expasy.org/protparam/).

### Chromosome distribution

The chromosomal positions of *PfMADS* genes were determined using genome annotation files. TBtools software was used to map the positions of *PfMADS* on chromosomes, ranging from short-arm to long-arm telomeres. Gene replication events in *PfMADS* were analyzed and identified using TBtools software.

### Motif analysis

GSDS2.0 (http://gsds.gao-lab.org/index.php) was used to analyze the gene structure of *PfMADS*, obtaining the positions and numbers of introns and exons. The conserved motifs of PfMADS proteins were predicted using MEME (http://meme-suite.org/meme/). Finally, visual phylogenetic analysis was performed uniformly using TBtools software.

### Construction of multiple sequence alignment and comparative phylogenetic tree

MEGA11 software was utilized to conduct a multiple sequence alignment analysis of the identified PfMADS and AtMADS protein sequences. The neighbor-joining (NJ) method was used to construct the phylogenetic tree, with the bootstrap test value (Bootstrap) set at 1000. The final visualization was performed using the Evolview website (https://evolgenius.info//evolview-v2).

### Gene replication and selection pressure analysis

The Multiple Collinearity Scan tool kit (MCScanX) in TBtools was used to examine segmental duplication events in the genome of *P. frutescens*. Additionally, it was used to calculate non-synonymous (Ka) and synonymous substitution (Ks) values for the segmental duplication gene pairs. The gene duplications were dated using the formula T = Ks/2r (Wu et al., 2017). The synteny relationship between genomes of the three species (*Perilla frutescens* and *Arabidopsis*, *Perilla frutescens* and *Salvia japonica Thunb.*) was analyzed using MCScanX, and the mutual homologous gene pairs were counted. Genomic data for *Salvia japonica Thunb.* were downloaded from NCBI (https://www.ncbi.nlm.nih.gov/assembly/?term=Salvia+farinacea).

### Expression profile and correlation analysis of *PfMADS* genes in different tissues

This study used *P. frutescens* seed transcriptome gene data, already available in the laboratory, to examine the expression profiles of *P. frutescens* seeds at three different stages (10d, 20d, 30d after flowering). Transcriptome data for roots, leaves, and buds were obtained from the NCBI *P. frutescens* SRA database (https://www.ncbi.nlm.nih.gov/sra/?term=Perilla+frutescens+%28L.). The TBTools software was used for expression profile cluster analysis. To investigate the regulatory role of the PfMADS in the expression of key genes related to lipid and flavonoid synthesis, partial gene sequences involved in these processes from NCBI. To clarify the specific relationship between MADS and these processes, we extracted the FPKM values from the gene transcriptome data. Correlation analysis was then conducted using the OmicsShare online tools (https://www.omicsshare.com/tools/Home/Soft/getsoft).

### Analysis of cis-acting elements in promoter sequences

The cis-acting elements in the 2000 bp promoter region upstream of *PfMADS* family members were analyzed using PlantCare (http://bioinformatics.psb.ugent.be/webtools/plantcare/html/)(Lescot et al., 2002), and the upstream promoter region was extracted for sequence analysis using TBTools.

### Plant material and low temperature and drought stress treatments

The plant material used in this experiment was the *P. frutescens* species ‘Zisu Jin No.2’, which was planted at the Agricultural Crop Station of Shanxi Agricultural University in April 2021. We selected ‘Zisu Jin No.2’ seeds with intact grains. Once the seedlings had grown to six true leaves, we carefully selected those that exhibited similar growth pattern. These selected seedlings were then subjected to cold temperature and drought stress treatments (1) The plants were grown at 4°C with a photoperiod of 16h light/8h dark. (2) To water the seedlings, PEG-6000 was added to the nutrient solution to create a 20% PEG Hoagland nutrient solution. Samples were collected in triplicate at the above three stress conditions. The seedling samples were immediately frozen in liquid N_2_ and stored at −80°C for subsequent RNA extraction.

### Genetic transformation of *Chlamydomonas reinhardtii*

*Chlamydomonas reinhardtii PADang.CC849* was utilized for genetic transformation in this study. A single algal colony of *Chlamydomonas reinhardtii* was carefully selected and inoculated into liquid TAP medium for culture activation. The culture conditions included a light intensity of 50 μmol·m^−2^·s^−1^, a 12-hour light-dark cycle, static culture at a temperature of 25 ± 1°C, and shaking the culture 4-5 times a day.

The E. coli strain DH5α and plant overexpression vector pHR13 used in this experiment are both stored at the Institute of Molecular Agriculture and Bioenergy of Shanxi Agricultural University.

The *PfMADS47* gene sequence was sent to Suzhou Jinweizhi Biotechnology Co., Ltd. Using *Chlamydomonas reinhardtii* as the target species, the expression vector pHR13-*PfMADS47*YH was constructed following codon optimization of the target sequence.

The bead-beating method (Wang C et al., 2017) was employed to genetically transform the wild-type strain CC849 of *Chlamydomonas reinhardtii*. Subsequently, the transgenic algal strains were obtained, and the algal biomass was collected for DNA and RNA extraction. PCR detection was performed at the gene and transcript levels to screen for positive transgenic algae strains.

### DNA and RNA extraction and qRT-PCR

The CTAB method was used to extract DNA from *Chlamydomonas reinhardtii* (Sun et al., 2016). Total RNA was extracted from samples using the EASY spin Plant RNA Rapid Extraction Kit (RN09, Beijing Adelaide Biotechnology Co., Ltd.). The cDNA was synthesized by reverse transcription using StarScript II RT Mix with gDNA Remover (A224, Beijing Kangrun Chengye Biotechnology Co., Ltd.), diluted at 1:10 dilution with RNase-free water, and stored at -20°C for subsequent qRT-PCR analysis. qRT-PCR amplification primers were designed based on the CDS sequences of low temperature and drought stress-related PfMADS genes (**Table S1**), and cDNA from different treatments was used as the template with the TB Green®Premix Ex Taq™ (Tli RNaseH Plus) kit [TaKaRa, Bao Ri Doctor Biotech (Beijing) Co., Ltd] to prepare the qRT-PCR reaction mixture: 5.0 μL TB Green Premix Ex Taq, 0.5 μL cDNA, 0.2 μL forward primer, 0.2 μL reverse primer, and 0.4 μL ddH_2_O, for a total volume of 10 μL. A TM real-time fluorescence quantitative PCR instrument was used for qRT-PCR amplification (DLAB-Accurate 96-X4). The amplification reaction was performed in three steps: 95°C for 2 min; 95°C for 30 s, 59°C for 30 s, and 72°C for 30 s, for 40 cycles. Three biological replicates and three technical replicates were established for each sample. The relative expression of *PfMADS* gene under low temperature and drought stresses was calculated by the 2^-ΔΔCT^ method using *Pfactin* as an internal reference gene.

### Nitrogen stress treatment of algal transformants

Algal cells cultured to the logarithmic growth phase were centrifuged at 5000 rpm for 3-4 minutes. The algal cells were collected and resuspended in a nitrogen-deficient medium. The uniform initial OD_680_ value was 0.8.

### Growth curve measurement

Shake the algal suspension evenly in the ultra-clean workbench and pipette 3 ml of each sample. Use the UV a spectrophotometer to measure the absorbance (OD_680_nm) of the algal suspension at a wavelength of 680nm. Changes in OD_680_nm indicates cells growth. Three replicates were set up, and each replicate was measured three times.

### Pigment extraction and analysis

Aliquots of 15 mL of the algal culture were harvested by centrifugation at 3000 g for 5 min. The resulting pellets were then re-suspended in 2 mL of acetone for pigment extraction. After vortexing for 30 minutes at maximum speed, the pigment extracts were collected by centrifugation at 13,000 g for 10 minutes. The extracts were evaporated under nitrogen gas and redissolved in acetone. Pigment analysis was performed using a Waters 2695 HPLC system equipped with a Waters Spherisorb 5 μm ODS2 4.6 × 250 mm analytical column (Waters, Milford, MA, USA), following the protocols previously described.

### Lipid extraction and analysis

Lipid extraction from *C. reinhardtii* was performed following the protocol described by Liu et al. (2016). In brief, algal samples were harvested by centrifugation at 3000 g for 5 minutes. The resulting cell pellets were then resuspended in 2 mL of methanol containing 0.05% butylated hydroxytoluene. After a 30-minute incubation, 4 mL of chloroform was added and the mixture was vigorously vortexed for another 30 minutes. Subsequently, 1.5 mL of 0.75% NaCl solution was added to facilitate phase separation. The lower organic phase containing the lipids was transferred into a new glass tube. The organic phase was then evaporated under nitrogen gas, and the remaining lipids were redissolved in chloroform for further quantification.

## Results

### Identification and physicochemical characterization of *PfMADS*

**Figure 1:**
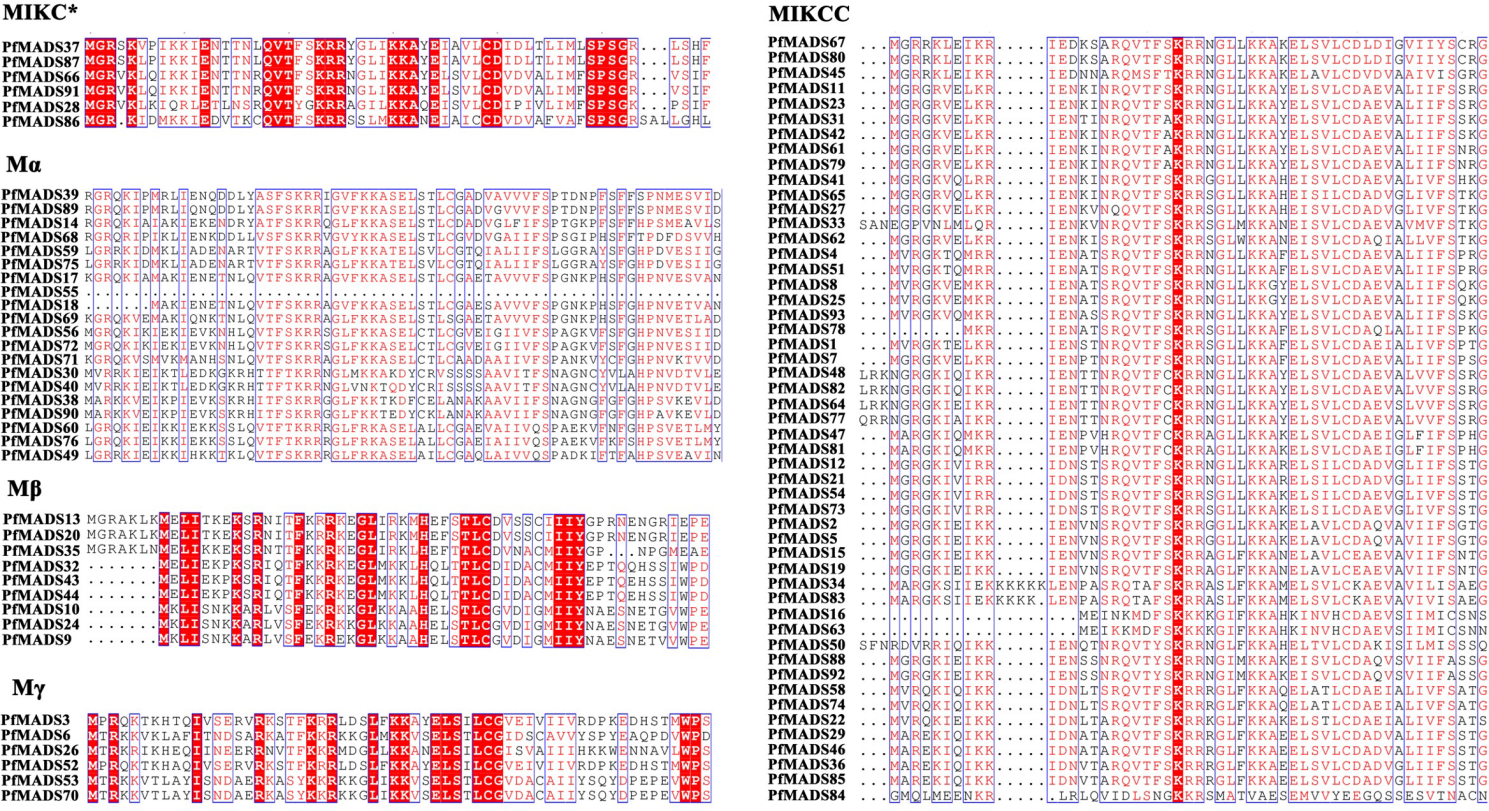
Multiple sequence alignment of the MADS domain from PfMADS. The red boxes indicated the higher conserved domains contained in each subfamily from PfMADS.

After removing redundant sequences, 93 *PfMADS* genes were finally identified in the genome of *P. frutescens*. Their characteristics, including gene locus ID, chromosomal start and end positions, member classification, protein sequence length (SL), molecular weight (MW), and isoelectric point (pI), were further specified. Statistical analysis showed that the length of the PfMADS proteins ranged from 64 to 380 amino acids, averaging of 236 amino acids. Their molecular weights ranged from 7.36 kDa to 43.53 kDa. The isoelectric points (pI) of the PfMADS proteins ranged from 4.58 to 10.4 (**Table S2**). To investigate the evolutionary relationships among PfMADS protein sequences, multiple protein sequence comparisons were performed for 93 PfMADS proteins (**Figure 1**). A highly conserved amino acid sequence, which spans approximately 30 to 60 amino acid residues, is present in different types of MADS domains (**Table S3**). These genes belong to the MADS-box gene family of *P. frutescens* and will be used in future research.

### Chromosome distribution and covariance analysis of *PfMADS*

**Figure 2:**
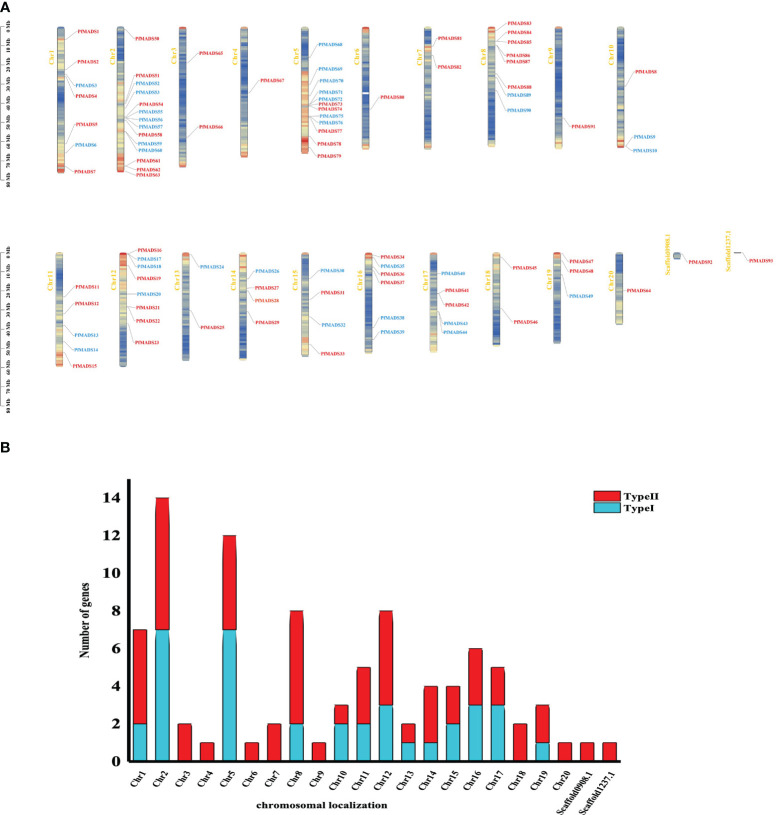
Analysis of the location of *PfMADS* on chromosomes. **(A)** Chromosomal distribution of *PfMADS* genes. The position of every *PfMADS* gene can be determined using the left scale. **(B)** a plot of the number and type of *PfMADS* genes per chromosome.

To investigate the genomic distribution of *PfMADS* genes, we performed a BLAST analysis against the released genome for *P. frutescens*. As a result, 93 *PfMADS* genes were mapped onto their corresponding chromosomes. It was observed that these genes are distributed unevenly across 20 chromosomes and 2 chromosomal segments. Chromosomes 2 and 5 contain the highest number of *PfMADS* genes, with 14 and 12 genes respectively. Conversely, the lowest number of *PfMADS* genes (one loci) were found on chromosomes 4, 6, 9, 20, scaffold0908.1, and scaffold1237.1(**Figure 2**). It is worth noting that the *PfMADS* gene family consists of 52 members from the AA subgenome and 41 members from the BB subgenome (**Table S4**). This discrepancy in numbers may have resulted from gene loss during the evolution of polyploidy.

**Figure 3:**
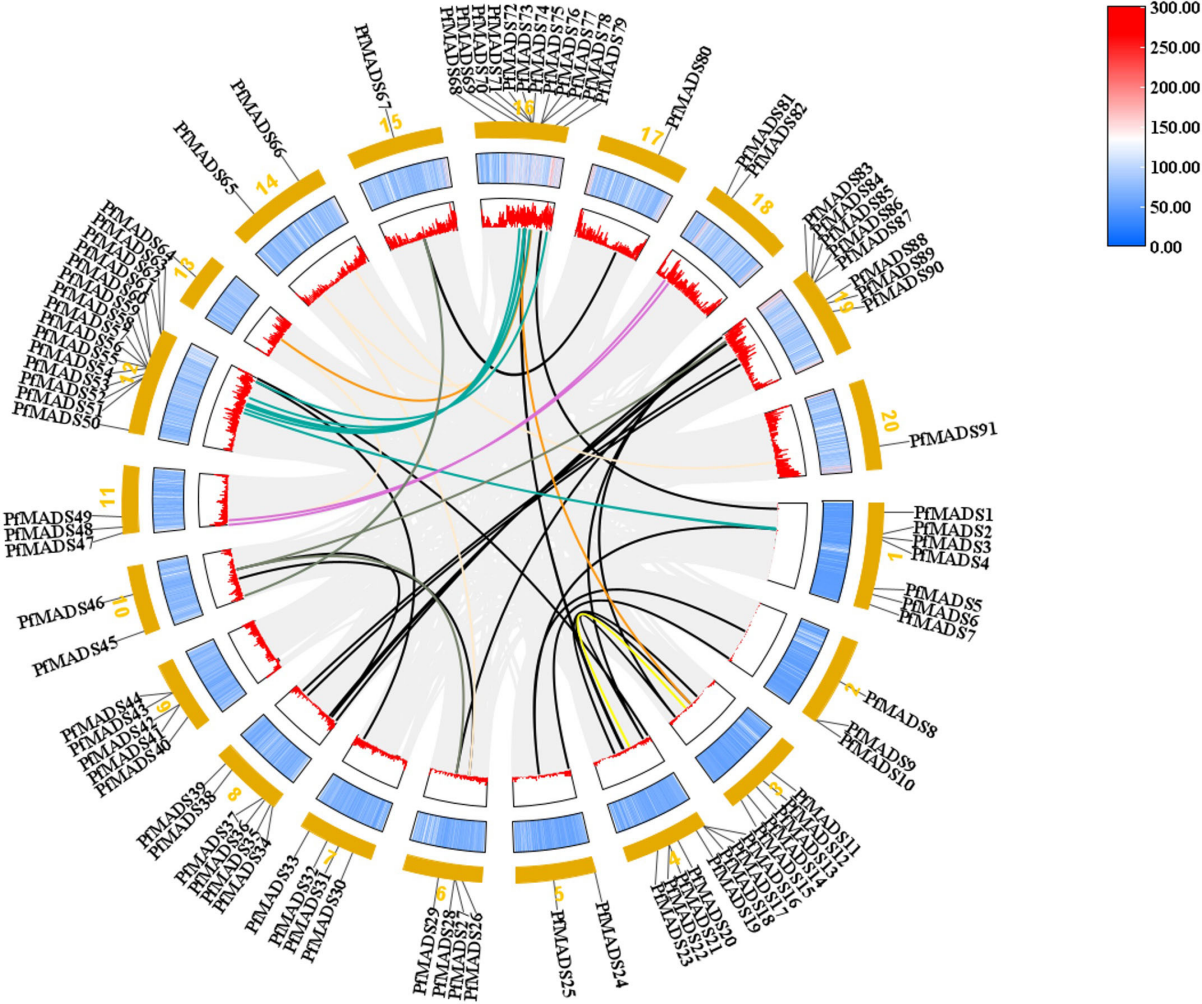
The intrachromosomal segmental duplication map of the MADS-box genes in *P. frutescens*. Colored lines inside the circle represent duplication inside the different chromosomes.

The replication events of *PfMADS* genes were analyzed at the genome-wide level by TBtools. The results revealed a significant homology between the *PfMADS* genes on different chromosomes (**Figure 3**). A total of 50 pairs of replication genes were counted (**Table S5**), all of them belonged to whole-genome duplication events, with 23 pairs having been duplicated only once. The remaining duplicated gene pairs had 2 to 4 copies each. To investigate whether these homologous genes underwent selection pressure, synonymous (Ks) and nonsynonymous (Ka) substitution rates were calculated for the identified gene pairs using TBtools, followed by the calculation of the Ka/Ks ratio to determine whether selection pressure acted on the PfMADS proteins (Zhang et al., 2017). Interestingly, the ω values for Ka/Ks of 47 gene pairs were less than 1, indicating that purifying selection has occurred for these gene pairs (**Table S5**).

### Phylogenetic analysis of PfMADS protein sequences

**Figure 4:**
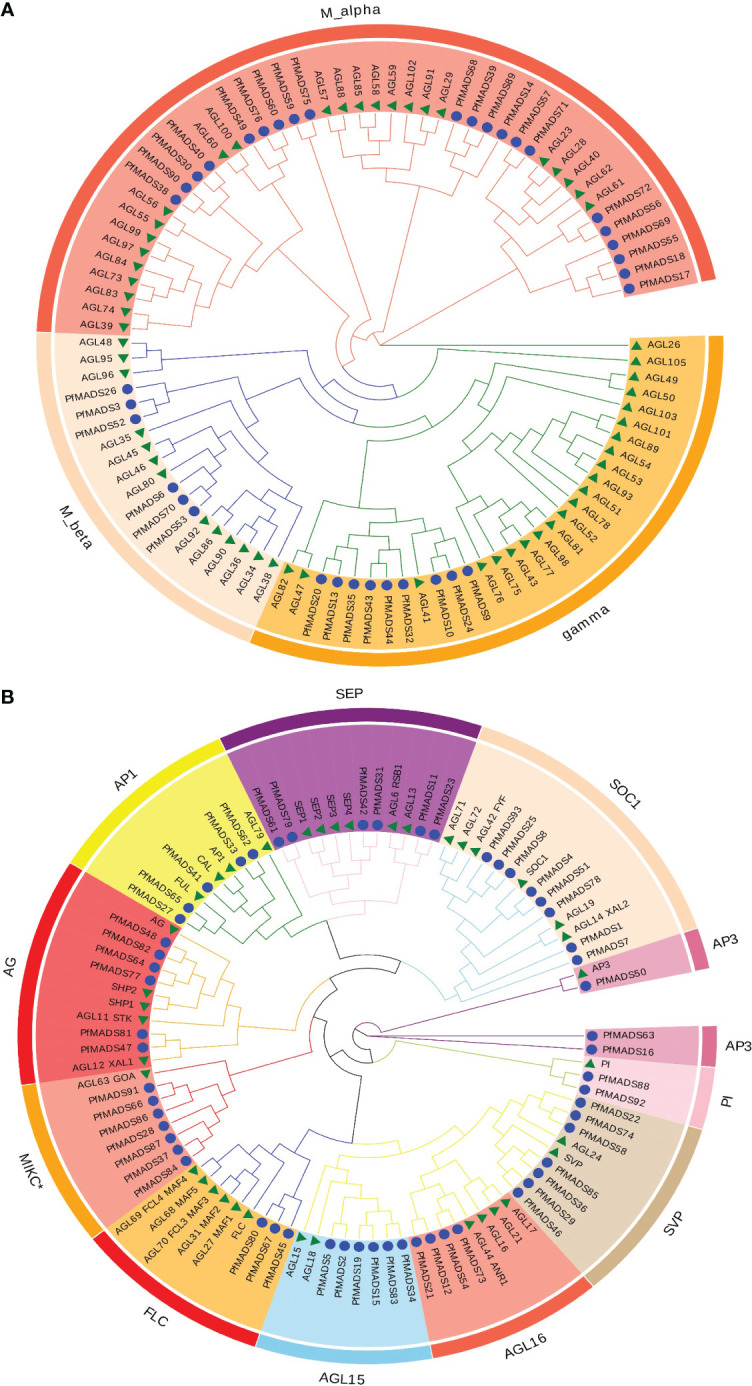
Classification of the *P. frutescens* MADS-box subfamilies. Specific-lineages are indicated by colors and bracketing. **(A)**, the type I lineages contain Mα, Mβ, and Mγ; **(B)**, the type II lineages contain MIKC* and MIKC^C^ (all other type II subfamilies except MIKC*).

To identify the phylogenetic relationship of PfMADS proteins, a phylogenetic analysis was conducted on MADS-box protein sequences from *P. frutescens* and *Arabidopsis* (**Figure 4**). Based on the grouping standards of AtMADS proteins, PfMADS proteins were classified into two primary types (Type I and Type II), with 37 PfMADS in Type I and 56 PfMADS in Type II. The PfMADS in Type I were further classified into three subfamilies (Mα, Mβ and Mγ) containing 22,9, and 6 members respectively. Moreover, Type II is further divided into AG, FLC, SEP, SVP, AP1/FUL, AP3, SOC1, PI, and MIKC* subfamilies. The numbers of their corresponding PfMADS proteins are 4, 4, 6, 7, 5, 3, 8, and 2, respectively. Compared to *Arabidopsis*, *P. frutescens* has a greater number of Type II members, although the total number (108 in *Arabidopsis*) is lesser. This finding suggests that the evolutionary pattern of Type II *PfMADS* genes is highly conserved. It also implies that Type II *PfMADS* genes might be involved in multiple biological functions. According to the phylogenetic tree, the MADS-box proteins from *P. frutescens* and *Arabidopsis*, which belong to the same classification, did not cluster together. Instead, they formed separate branches with similar evolutionary relationships. This observation can largely explain the variations in the evolutionary patterns of the MADS-box gene family among different species.

### Homology analysis of MADS-box genes in *P. frutescens* and two plants

**Figure 5:**
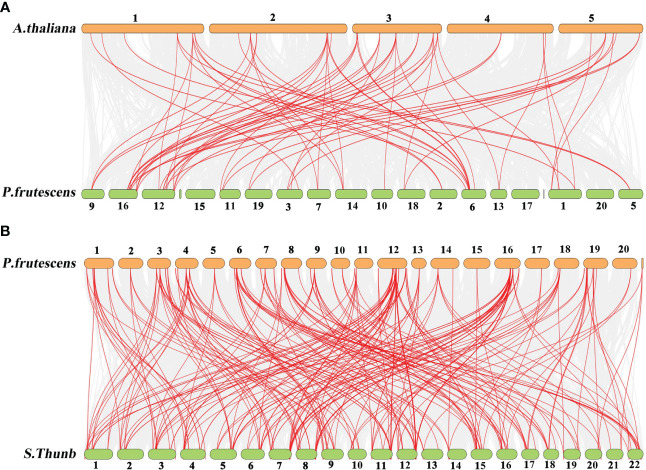
Synteny analysis of *PfMADS* genes between *P. frutescens* and two plant species (*A. thaliana* and *Salvia japonica Thunb.*). **(A)**, *P. frutescens* and *A. thaliana* ***(*B*)*** *P. frutescens* and *Salvia japonica Thunb.* The chromosome number was indicated at the top of every chromosome.

The comparative synteny maps of two genomes (*P. frutescens* vs *A. thaliana*, and *P. frutescens* vs *Salvia japonica Thunb.*) were created to explore the origin and evolution of *PfMADS* genes (**Figure 5**). Orthologous relationships were detected between 93 *PfMADS* genes and 108 *AtMADS* genes, and then 80 orthologous *MADS-box* gene pairs were identified accordingly, with most of them located on the syntenic loci on *A. thaliana* and *P. frutescens* chromosomes (**Figure 5A**). Multiple *PfMADS* genes were identified as putative homologs of the *AtMADS* genes. For instance, *PfMADS48*, *PfMADS77*, and *PfMADS82* were identified as orthologs genes of AG. Similarly, the orthologous relationships were also present between 93 *PfMADS* genes and 117 *ShMADS* genes, and the corresponding 245 orthologous MADS-box gene pairs were established, with many found on the syntenic loci in the chromosomes of *P. frutescens* and *Salvia japonica Thunb.* (**Figure 5B**).

### Conserved motifs and gene structure analysis of *PfMADS*

**Figure 6:**
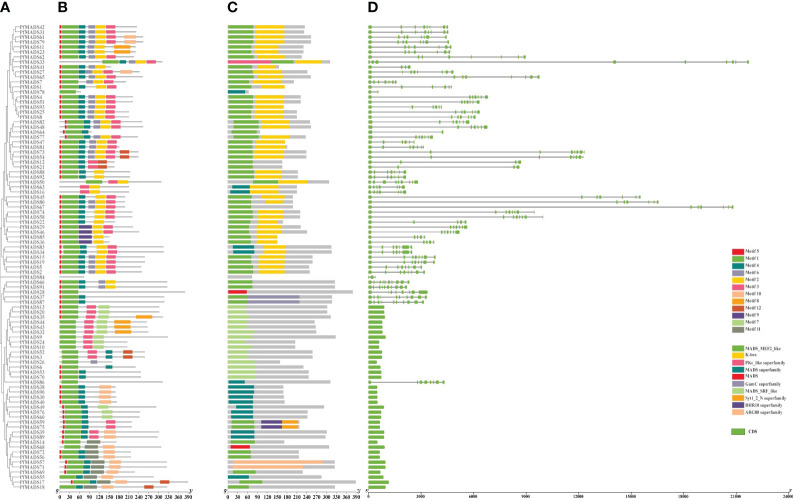
Gene and protein structures of the *MADS-box* gene families in *P. frutescens*. **(A)** Phylogenetic tree of *P. frutescens* MADS-box genes; **(B)** Motifs of MADS-box proteins. **(C)** Conserved domains of MADS-box proteins; **(D)** Exon-intron structures of MADS-box genes.

To reveal the functional regions of PfMADS proteins (**Figure 6A**), conserved motifs were predicted by the MEME (Multiple Em for Motif Elicitation) program, and a total of 12 motifs were detected in 93 PfMADS proteins (**Figure 6B**). In accordance with the results of conservative motif analysis, Motifs 1 was detected in all PfMADS proteins. Moreover, Motifs 2 were predicted in most Type II except for Type I. Therefore, compared to Type I, the structure of Type II PfMADS proteins is relatively complex.

The PfMADS protein sequences were analyzed using MEGA software to perform multiple sequence alignment analysis and visual processing (**Figure 6C**). The results indicated that the PfMADS protein domains are remarkably conserved, with all 93 members containing MADS domains. Generally, the MADS domain of Type I is classified as SRF_like, while the domain of Type II is classified as MEF2_like.

Gene structure analysis of 93 *PfMADS* genes (**Figure 6D**) revealed that the number of exons in members of the *PfMADS* gene family varies from 1 to 11. Additionally, the number, distribution, and length of exons in *PfMADS* genes within the same subfamily exhibit similarities. The gene structure of M-type (Type I) *PfMADS* does not contain any introns, and the length of these genes is typically short, usually less than 1kb. On the other hand, Type II *PfMADS* genes have an average of 7 exons and introns in their gene structure, and they tend to be longer, mostly exceeding 3kb. These results established a significant molecular biology foundation for the functional investigation of *PfMADS* genes.

### Expression patterns of *PfMADS* genes in different tissues

**Figure 7:**
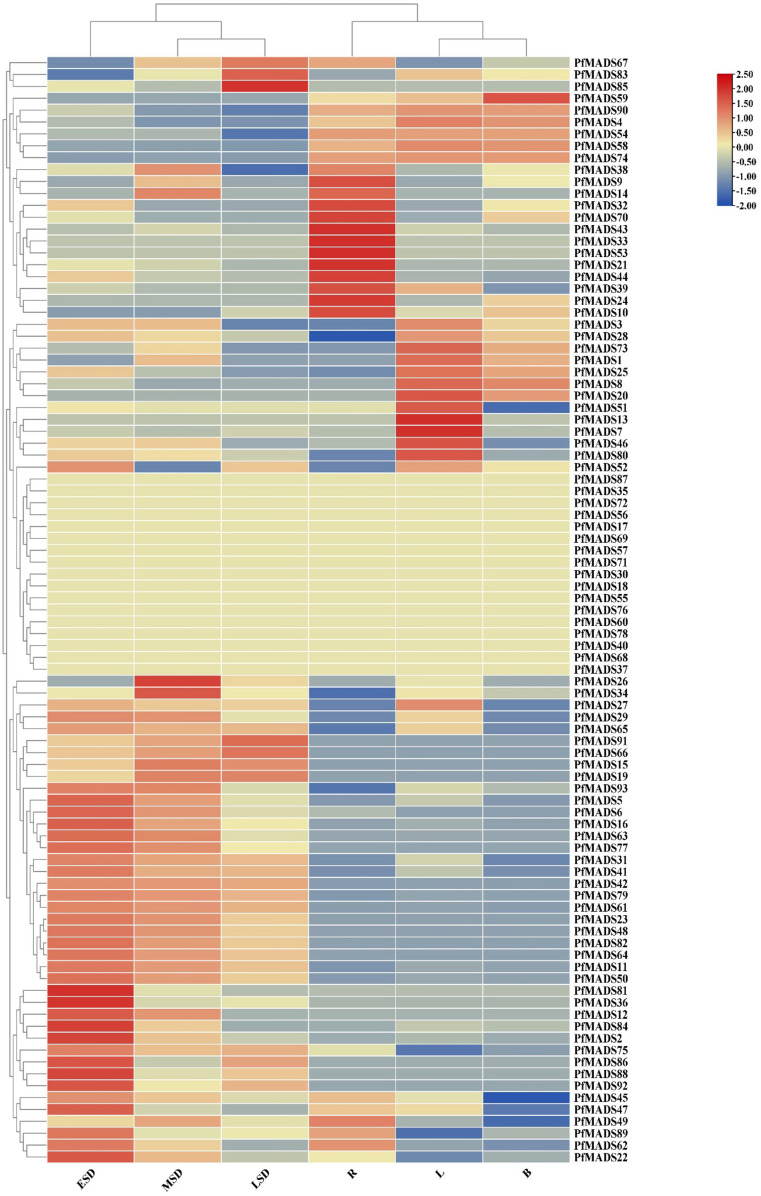
Expression profiles of *PfMADS* genes during six developmental stages of *P. frutescens*. The expression levels of *PfMADS* genes were showed by different colors on the right scale. ESD to LSD respectively indicated 10,20 and 30 days after full blooming. R, roots; L, leaves; B, bud.

The gene expression pattern can provide essential information for determining the biological function of a gene. In order to study the function of the *PfMADS* genes, the data (FPKM) of *P. frutescens* in the public database were used to conduct expression analysis on 6 tissues. As depicted in **Figure 7**, genes such as *PfMADS59/90/58/74* are highly expressed in flower buds, among which *PfMADS59/90* belongs to the Mα subfamily and *PfMADS58/74* belongs to the *SVP* subfamily. The found in apple that heterologous expression of *MdDAMb* and *MdSVPA* genes in ‘Royal Gala’ apple plant resulted in delayed buds and structural changes (Wu et al., 2017). Therefore, *PfMADS58/74* in *P. frutescens* flower buds may be involved in the differentiation of floral meristems. **Figure 7** shows that there are some important seed-specific or seed-dominant genes. For example, *PfMADS85*/*83*/*67*/*26*/*34*/*91*/*66*/*15*/*19* are mainly expressed in later stages of seed development, and might be involved in development and metabolism of later stages of *P. frutescens* seeds. Many genes such as *PfMADS81*/*36*/*12*/*84*/*2*/*75*/*86*/*88*/*92* and so on are mainly expressed in early stages of seed development, and might be involved in development and metabolism of early stages of *P. frutescens* seeds. Overall, more than 80% of *PfMADS* genes exhibit expression in these tissues, and members of the same subfamily demonstrate similar expression patterns. However, there are variations in the levels of expression. Type II *PfMADS* is predominantly expressed in seeds and leaves, while certain genes exhibit exclusive expression in roots and leaves. We speculate that *P. frutescens* likely has a considerable number of type II genes that function in various tissues.

### Expression analysis of several *PfMADS* genes in different colored leaves of *P. frutescens*

**Figure 8:**
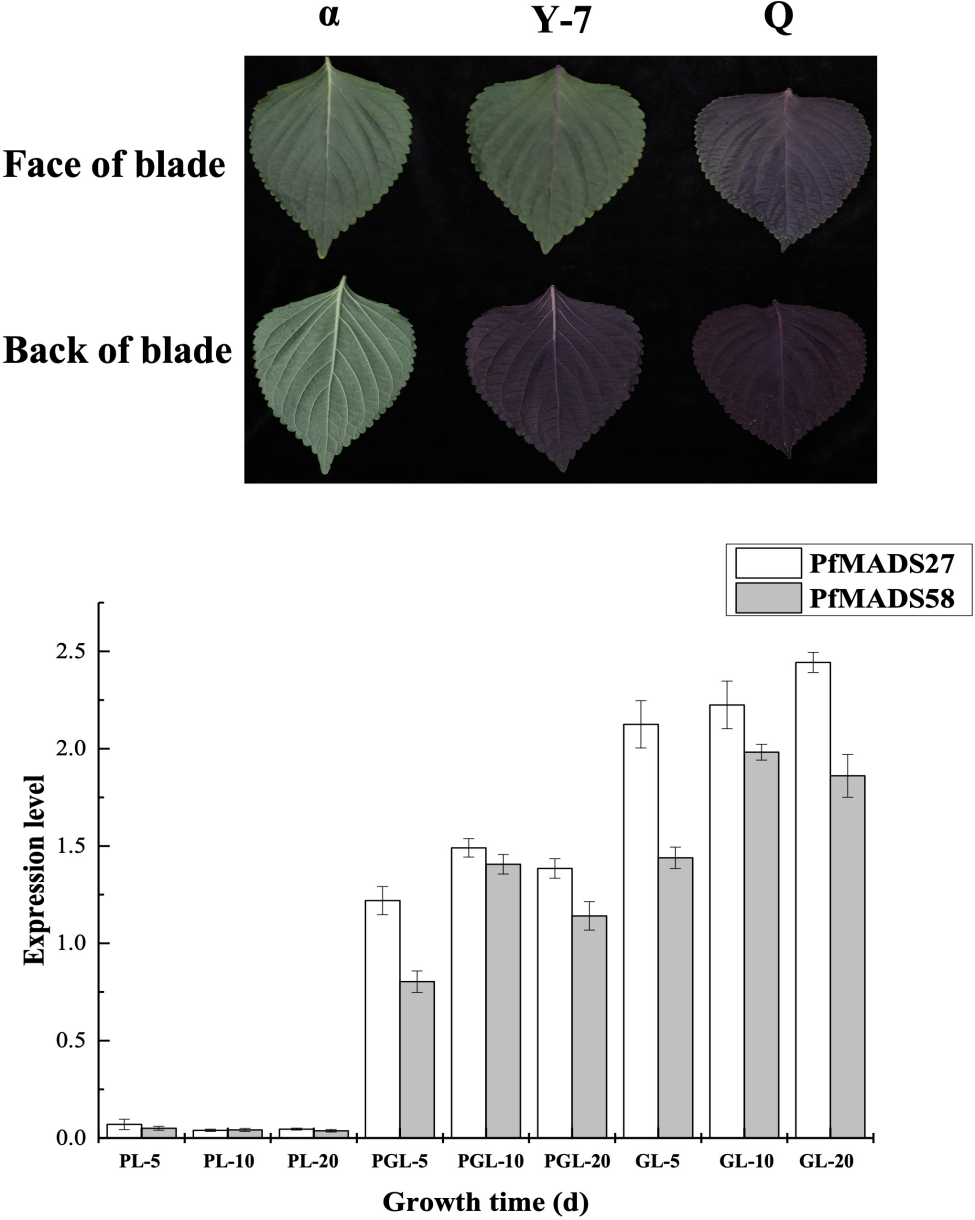
Expression profiles of *PfMADS* genes in different color leaves during three developmental stages of *P. frutescens*. PL, purple leaf; PGL, half purple, half green leaf; GL, green leaf. The *P. frutescens* seedlings were sampled on the 5^th^,10^th^ and 20^th^ day after the second true leaf. Data are means ± SE calculated from three biological replicates.

Flavonoids, including anthocyanins, found in *P. frutescens* leaves have been the subject of extensive research. In this study, we identified several *PfMADS* genes from the transcriptome data of *P. frutescens* leaves and analyzed their expression at different stages of leaves with varying colors. The results from **Figure 8** indicate that *PfMADS27* and *PfMADS58* were barely expressed in purple leaves, while they were expressed in green leaves. Additionally, as the color of the leaf transitions from purple to green, there is a corresponding gradual increase in the expression level of *PfMADS27*. This suggests a possible involvement of *PfMADS27* in the synthesis process of flavonoids, particularly anthocyanins. However, further investigation is required to confirm this hypothesis.

### Multiple *PfMADS* genes may participate in the pathways of lipid synthesis and flavonoid metabolism

**Figure 9:**
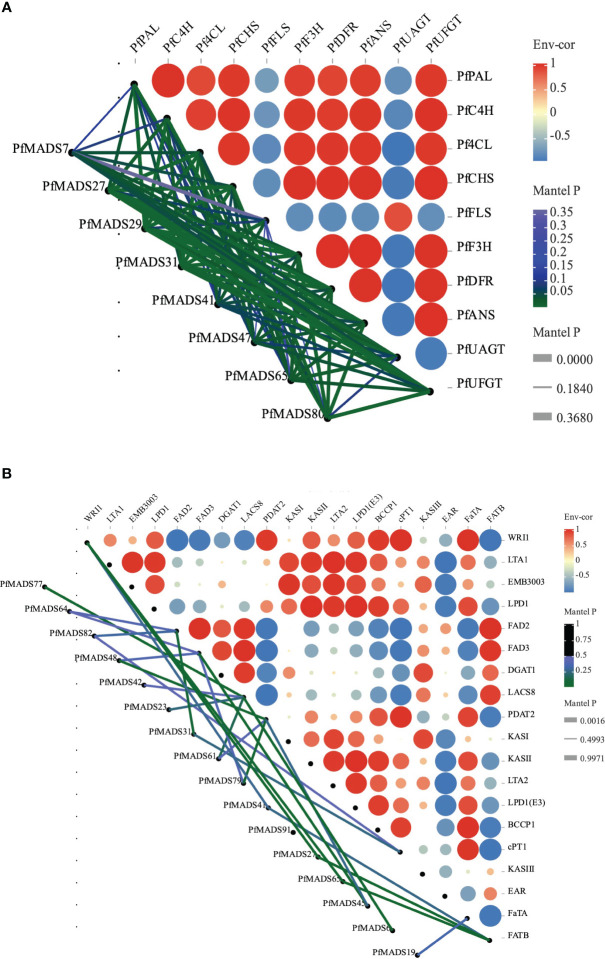
Correlations of expression patterns of *PfMADS* between other genes. **(A)** Correlations of expression patterns between *PfMADS* and the flavonoid metabolic related genes. Line thickness mapping absolute value of correlation, color mapping p value credibility. **(B)** Correlations of expression patterns between *PfMADS* and the lipid biosynthetic related genes. Line thickness mapping absolute value of correlation, color mapping p value credibility.

To explore the potential transcriptional regulatory mechanism of *PfMADS*, we performed correlation analysis of *PfMADS* genes related to oil accumulation and key genes related to flavonoid metabolism in seeds and leaves of *P. frutescens*. The results indicate that out of the 93 *PfMADS* genes, 9 are closely associated with important genes involved in lipid synthesis (**Figure 9A**). These findings suggest that these 9 genes can serve as potential candidates for further investigations into their functional roles. Eight *PfMADS* genes were selected to conduct correlation analysis with key genes in the flavonoid metabolism pathway (**Figure 9B**), and the results were consistent with those reported (Jiang et al., 2020). One of the genes, *PfMADS47*, is worth investigating as it is closely associated with lipid synthesis. However, further molecular-level verification, such as transgenesis, is necessary for functional confirmation.

### Analysis of cis-acting elements of the promoter of *PfMADS* genes

**Figure 10:**
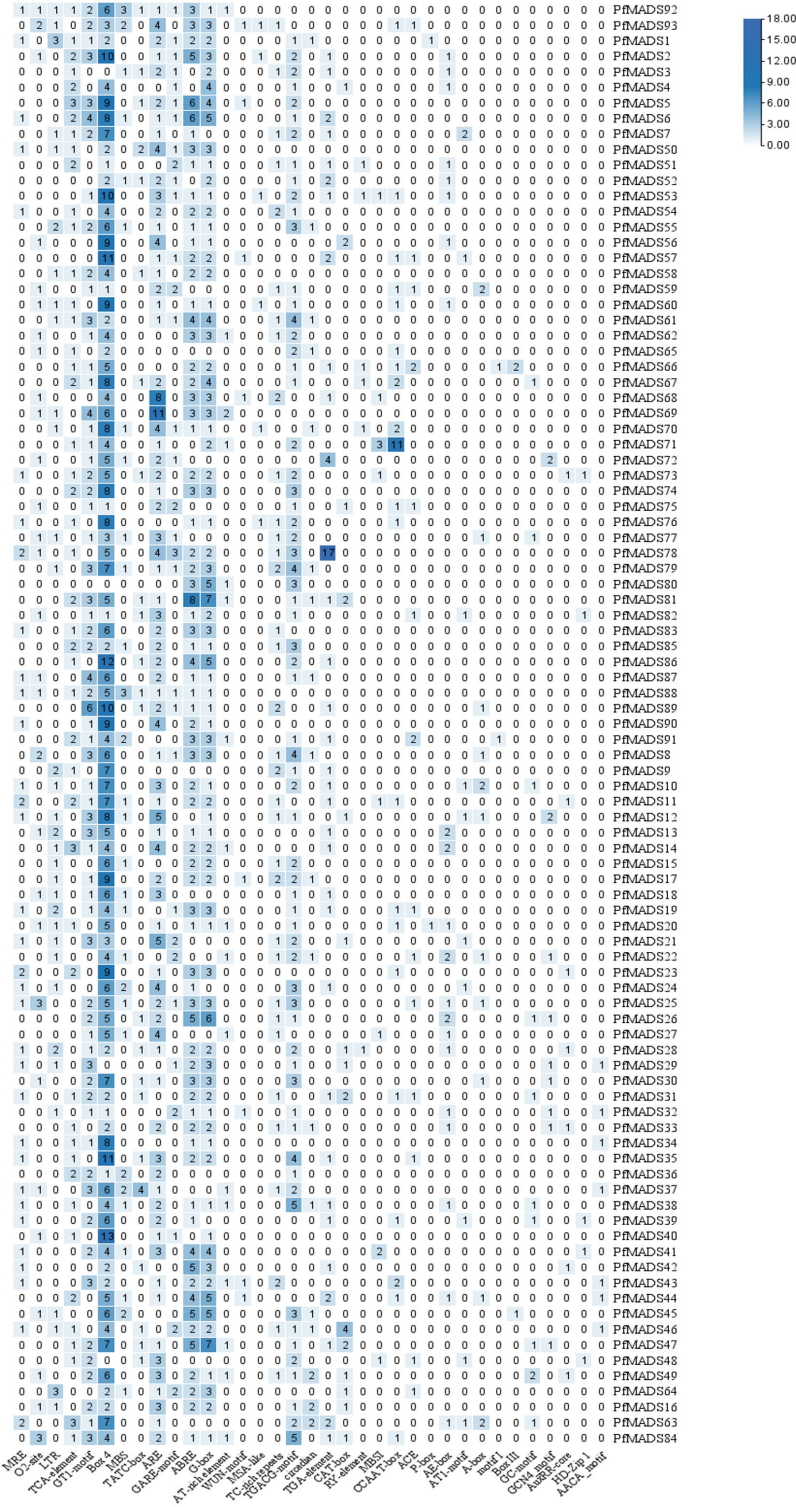
Cis-acting elements of *PfMADS* gene promoter from *P. frutescens*. The numbers in the box represent the number of cis-elements contained in each *PfMADS* gene.

To gain a deeper understanding of the biological processes and molecular regulatory mechanisms involving *PfMADS* genes, we conducted cis-acting element analysis on the 2000 bp promoter region upstream of the start codon of 93 *PfMADS* using PlantCare. More than 70% of *PfMADS* genes contained light-responsive elements and hormone-responsive elements in their promoters, as shown in **Figure 10**. This suggests that a majority of *PfMADS* genes may play a role in regulating life processes that are influenced by light or hormones. In addition, several MYB binding sites including MBS, MBSI, MRE and CCAAT-box associated with drought, photoresponse and flavonoid biosynthesis were identified in many *PfMADS* gene promoters, demonstrating that these PfMADS proteins may interact with MYB TFs to involve in regulation of drought stress response, photoperiod and flavonoid biosynthesis. The *PfMADS* gene family members potentially have significant roles in biological processes, including perilla hormone and stress responses. These findings provide valuable candidate genes for subsequent cloning and functional analysis.

### Expression analysis of related *PfMADS* genes under cold and drought stresses

Based on the analysis results of cis-acting elements in the promoters of *PfMADS*, six specific genes were selected. These genes, namely *PfMADS15/22/25/45/80*, and *93*, were found to contain both cold and drought stress response elements. Subsequently, the expression profiles of the six *PfMADS* genes were examined under drought (DS) and cold stress (CS).

**Figure 11:**
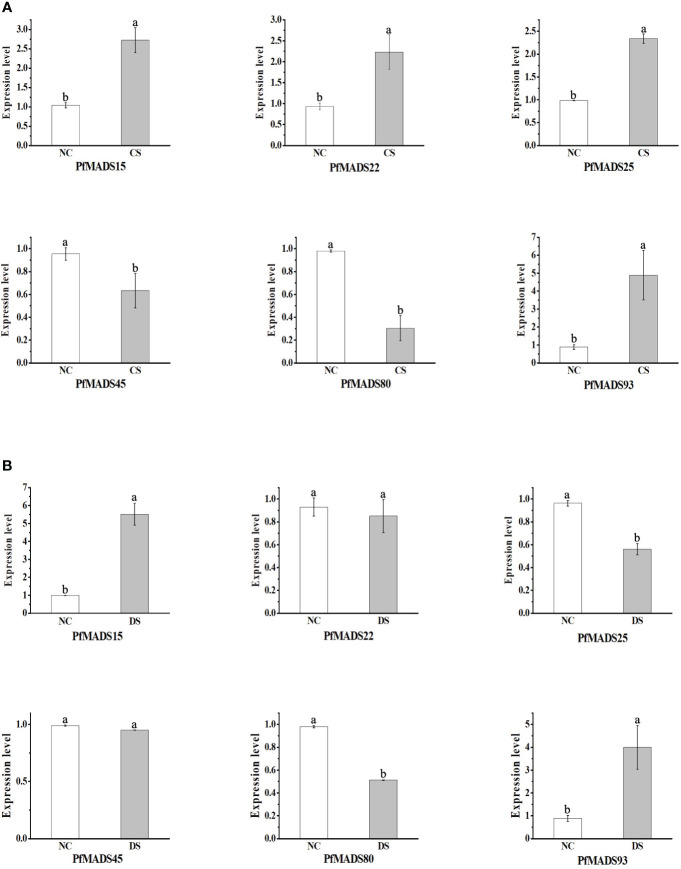
Expression profiles of some *PfMADS* genes under two stress conditions. **(A)** Expression profiles of some *PfMADS* genes in perilla seedlings under cold stress. CS, cold stress; NC, normal conditions. **(B)** Expression profiles of some *PfMADS* genes in perilla seedlings under drought stress. DS, drought stress; NC, normal conditions. Data are means ± SE calculated from three biological replicates.

As depicted in **Figure 11A**, the expression levels of *PfMADS22*/*25*/*45*/*80* genes were found to be lower under drought stress compared to the control group (NC). Among them, the relative expression of *PfMADS22* and *PfMADS45* were not significantly down-regulated and remained relatively unchanged. In comparison to the control group, the expression levels of *PfMADS25* and *PfMADS80* decreased by 42% and 48% respectively, suggesting that *PfMADS25* and *PfMADS80* may have a negative regulatory role in the response of *P. frutescens* to drought stress. Conversely, the relative expression of *PfMADS15* and *PfMADS93* increased by 5.5 times and 4.9 times severally. This suggests that these two *PfMADS* genes may have a positive regulatory role in the drought stress.

The expression levels of *PfMADS15*/*22*/*25*/*93* were all increased under cold stress (CS) (**Figure 11B**). However, the up-regulation of *PfMADS15*, *PfMADS22*, and *PfMADS25* was relatively small compared to the up-regulation of *PfMADS93*. It is predicted that these *PfMADS* genes play a positive regulatory role in the response of *P. frutescens* to cold stress. The relative expression of *PfMADS45* and *PfMADS80* decreased under cold stress, suggesting their involvement in the negative regulation of *P. frutescens* response to cold stress.

### Transformed strains of *PfMADS47* promoted oil accumulation under nitrogen stress

Microalgae has gained increasing attention as a valuable biological resource and a promising renewable bioenergy material (Ahmed et al., 2017). Previous studies have focused on enhancing oil production by genetically modifying *Chlamydomonas reinhardtii* (Wang et al., 2017; Wang et al., 2018; Zhang et al., 2019). In this study, we investigated the expression profile of the *PfMADS* genes in perilla seeds and its correlation with lipid metabolism genes. Based on these findings, we identified *PfMADS47* as a potential regulator of perilla lipid metabolism and constructed a transformation vector for *Chlamydomonas reinhardtii*. The constitutive expression vector pHR13-*PfMADS47* (**Figure S1A**) was introduced into algal cells using the glass bead method. Transgenic algal strains were successfully identified at the genomic and transcriptomic levels (**Figure S1**).

**Figure 12:**
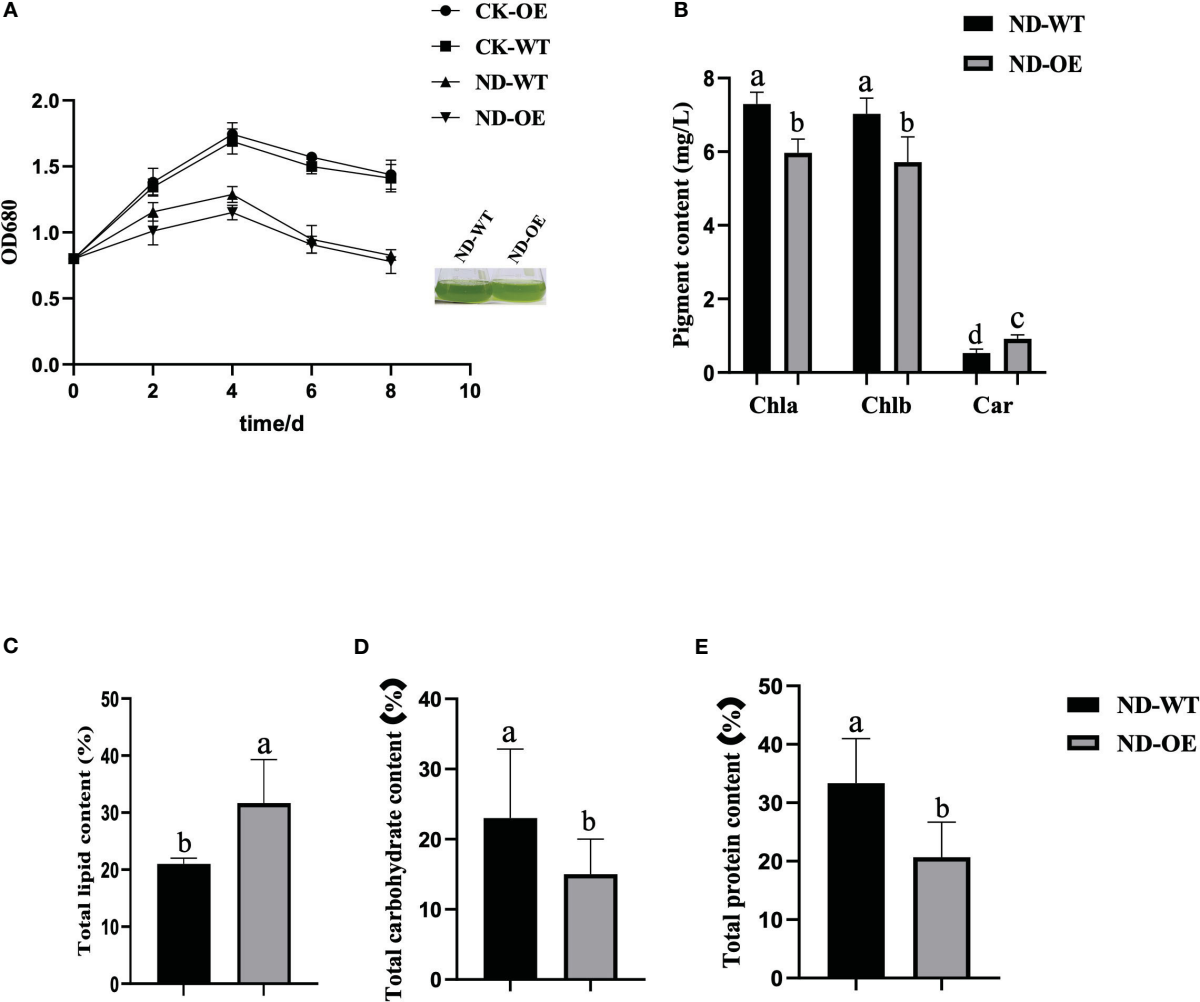
Physiological and biochemical indicators of algal cells under nitrogen stress. **(A)**. Growth curve. **(B)**. Pigment content. Chla, chlorophyll a; Chlb, chlorophyll b; Car, carotenoid. **(C)**. Total lipid content of algal cells. **(D)**. Total carbohydrate content of algal cells. **(E)**. Total protein content of algal cells. Columns with the same substance and the same indicator are marked with different lowercase letters to indicate significant differences (P<0.05).

Several studies have demonstrated that nitrogen stress can effectively induce lipid biosynthesis in *Chlamydomonas reinhardtii* and enhance total lipid accumulation (Yang et al., 2015). By subjecting the *PfMADS47* algal strains to nitrogen stress, we observed a 45% increase in the total lipid content of the transformed strains compared to the wild strains (**Figure 12C**). Conversely, the total carbohydrate and protein contents of the transformed algal strains showed a significant reduction of 43% and 49% respectively (**Figures 12D, E**). Moreover, the growth rate of the algal cells decreased under nitrogen stress (**Figure 12A**), accompanied by a decrease in photosynthesis (**Figure 12B**), which aligns with the findings reported by Wang. These findings suggest that the introduction of the *PfMADS47* gene in microalgae enhances oil production, redirects carbon sources from carbohydrate and protein synthesis pathways towards the oil biosynthesis pathway, and stimulates oil metabolism in algae.

## Discussion

The MADS-box gene family has been extensively studied and analyzed in various species. MADS-box transcription factors are widely found in animals, plants, and fungi, but the number and types of MADS-box genes differ significantly among species. The number of members and subfamily classification of MADS-box genes in lower plants, such as mosses (Henschel et al., 2002) and algaes (Tanabe et al., 2005; Nayar and Thangavel, 2021), are relatively small. Additionally, there have been no articles reporting the presence of type II genes containing K domains in microalgae. On the other hand, the number and types of MADS-box genes are abundant in higher plants, with 108 in *Arabidopsis thaliana*, 168 in tobacco, 160 in cabbage, and so on (Bai et al., 2019; Won et al., 2021). This suggests that MADS-box genes have undergone quantitative expansion during the evolution process in higher plants. The increased number of MADS-box genes in higher plants also indicates functional redundancy within this gene family. In conclusion, the expansion of MADS-box from lower plants to higher plants during the evolution process suggests that MADS-box is functionally redundant in higher plants.

In this study, a total of 93 *PfMADS* genes were identified (**Table S2**). The overall number is lower than that of diploid *Arabidopsis*, possibly due to the tetraploid genome of *P. frutescens*. Some studies have reported that the *P. frutescens* genome is of the AABB type. However, our *PfMADS* family comprises of 52 members from the AA subgenome and 41 members from the BB subgenome, indicating an unequal distribution (**Table S4**). It is speculated that this disparity may be attributed to gene loss resulting from the balanced exchange phenomenon between subgenome in the *P. frutescens* genome, as well as the enrichment of homologous substitutions in the proximal telomere regions of the chromosome (Zhang et al., 2021). It has been reported that during polyploidy events, species undergo chromosomal reorganization, which often leads to the loss of a significant number of genes (Akter et al., 2021). Furthermore, gene deletion disrupts the collinearity between genes. Extensive genetic separation and genome recombination further diminish the similarity to the ancestral species (Fang et al., 2012; Zhao et al., 2021). In addition, the MADS-box family members of two other species from the Lamiaceae family, namely the diploid sage (123 gene numbers) and the tetraploid salvia (131 gene numbers), were preliminarily identified (**Tables S6**, **7**). The results did not show a regular pattern, suggesting that there may have been fewer repeated events during the evolution of the ancestors of the Lamiaceae family. Furthermore, we observed that the number of *PfMADS* genes is similar to that in *Camellia chekiangoleosa* (89) and *Musa balbisiana* (97) (Lakhwani et al., 2022; Zhou et al., 2023), but higher than in cucumber (43), sesame (57), and pineapple (48) (Hu and Liu, 2012; Wei et al., 2015; Zhang et al., 2020). On the other hand, perilla has a lower number of genes compared to those in poplar (105) and tomato (131) (Leseberg et al., 2006; Wang et al., 2019). These species do not appear to be closely related, which suggests that complex historical events may have influenced their gene numbers. However, further investigation is required to determine the specific reasons (Wu et al., 2017).

Each subfamily of *PfMADS* exhibits a relatively conserved sequence of 30 to 60 amino acids, as shown in **Figure 1**. There are 37 Type I members distributed among 3 subfamilies, and 56 Type II members distributed among 11 subfamilies, as illustrated in **Figure 4**. The higher number of Type II genes in *P. frutescens* compared to *Arabidopsis* suggests that the Type II *PfMADS* genes, especially the MIKC^C^ subfamily, underwent similar gene duplication events during ancestral evolution as observed in *Arabidopsis*. This indicates that the MIKC^C^ genes may have faced stronger selection pressure during the evolutionary process, potentially impacting the environmental adaptability of *P. frutescens*. The distribution of *PfMADS* on chromosomes shows heterogeneity and clustering (**Figure 2**). Interestingly, Type I *PfMADS* tends to be distributed more in clusters, while Type II *PfMADS* are more uniformly distributed compared to Type I, which is consistent with the results of chromosome alignment analysis of MADS-box in *Arabidopsis* and rice (Nam et al., 2004). Specifically, a few regions with a higher density of *PfMADS* genes were observed on some chromosomes, such as chr2 and chr5, suggesting that there might be *PfMADS* genes hotspots in *P. frutescens*. According to some studies, gene families evolve and new functional genes are generated through tandem repeats and fragment repeats (Cannon et al., 2004). However, in the case of *PfMADS*, no tandem repeats were found, suggesting that the main driver of *PfMADS* gene family evolution and amplification in *P. frutescens* is fragment duplication (**Figure 3**).

In *P. frutescens*, motif 1 encodes the typical MADS-box TFs (SRF), which are extremely conserved in PfMADS (**Figure 6**). The K domain is a conserved domain in the MADS-box gene family. Typically, the K domain is found only in the MIKC^C^ subfamily and is represented by motif 2 in *P. frutescens*. (Kaufmann et al., 2005). MIKC-type MADS-box proteins, even without the K domain, retain the ability to bind DNA and function as full-domain proteins (Mattick, 1994). However, the lack of the K domain leads to functional impairment of the TaSEP1-A2 protein, as it prevents protein-protein interactions (Shitsukawa et al., 2007). These results indicate that the motifs present in PfMADS proteins vary significantly across different subfamilies. However, within the same subfamily, there are certain similarities in the types, numbers, and distribution of motifs in PfMADS proteins. This suggests that these genes may have distinct functions specific to their respective subfamilies (Wang et al., 2019).

Previous studies have demonstrated that exon-intron gene structures remain relatively conserved throughout evolution (Rogozin et al., 2003). Nevertheless, the gain or loss of introns plays a crucial role in generating variations in intron positions and in the emergence of novel genes (Rogozin et al., 2005; Roy and Penny, 2007). Different gene structures were observed in *PfMADS* genes. Type I genes lack introns (**Figure 6D**), which aligns with a finding suggesting that evolution may not only impact gene function, but also genetic structure (Babenko et al., 2004; Roy and Penny, 2007). Phylogenetic and gene structure analyses revealed that Type II genes within the same subfamily exhibit similar exon-intron structures. Furthermore, the gene structures of *PfMADS* genes correlate with the grouping of their phylogenetic trees. Significant differences exist between different subfamilies, whereas the distribution of gene exons and introns within the same subfamily is similar. Based on these findings, it is suggested that the gene structures of *PfMADS* gene members belonging to the same subfamily are closely linked to phylogenetic evolution. Additionally, it is plausible that the varied gene structures may contribute to their distinct functions (Parenicová et al., 2003).

The gene expression pattern can provide crucial insights into determining the biological function of a gene. In this study, we conducted transcriptome analysis of MADS-box genes in six different tissues of *P. frutescens*. The data revealed that the expression of *PfMADS* is specific to each tissue, which aligns with their respective gene functions (**Figure 7**). The alanine content of *P. frutescens* seeds is considerably higher compared to other major oil crops. In this study, we utilized the transcriptome data of *P. frutescens* seeds to investigate the expression pattern of *PfMADS* during three developmental stages. In the expression profile of early seed development, the highly expressed *PfMADS12*/*36*/*75*/*81*/*88*/*92* genes are classified into subfamilies such as *AG*/*Mα*/*AGL11*/*AGL15*. By referring to the *Arabidopsis* Information Network (https://www.arabidopsis.org/browse/genefamily/MADSlike.jsp), it is suggested that these genes may play a role in the development of endosperm, ovule, and seed coat. On the other hand, genes like *PfMAD15* (*AGL15*), *PfMADS67* (*FLC*: related to vernalization), and P*fMADS91* (*AGL63*) show high expression in the late stages of seed development (Xi et al., 2020). It is speculated that these genes may be involved in the process of seed germination. Additionally, we examined the correlation between *PfMADS* members and key genes involved in lipid synthesis, as illustrated in **Figure 9A**. It is worth noting that *PfMADS47*、*48*、*64*、*77*and *82* exhibit high expression levels during the early stages of seed maturation and show strong correlation with key genes involved in lipid synthesis. Coincidentally, all of these genes belong to the AG subfamily, which supports the findings of Zhang et al. (Zhang et al., 2017). In oil palm protoplasts and callus overexpressing *EgAGL9*, there was a significant reduction in the total fat content and unsaturated fatty acid content, including oleic acid, linoleic acid, and linolenic acid (Zhang et al., 2022). To further investigate, we overexpressed *PfMADS47* (AG subfamily) in *Chlamydomonas reinhardtii*, a genetically transformed model plant. Our research reveals that *PfMADS47* plays a role in promoting lipid accumulation in microalgae and actively participates in the lipid synthesis and metabolism pathway. These findings provide a foundation for future genetic engineering approaches aimed at enhancing the oil content of *P. frutescens* seeds.

*P. frutescens* leaves have multiple uses as medicinal materials, vegetables, and spices. They are known to contain a diverse range of chemical components, including flavonoids and polysaccharides (Fiedor and Burda, 2014; Holt et al., 2005; Li et al., 2022). The flavonoid compounds in plants have distinct functions in response to specific developmental stages or abiotic conditions. They serve as the primary defense mechanism against ultraviolet rays and pathogens (Kawser Hossain et al., 2016; Tejada et al., 2018). The expression of several genes in leaves of different colors was analyzed by qRT-PCR, and a special gene *PfMADS27* was found, which was speculated to be involved in the process of flavonoid synthesis (**Figure 8**). In a study of flavonoid anabolic pathways in *P. frutescens* leaf slices (Jiang et al., 2020), eight *PfMADS* genes were identified as possibly involved in the flavonoid anabolic pathway in *P. frutescens*. We further verified this by conducting a correlation analysis (**Figure 9A**). The results demonstrated a strong correlation between the *PfMADS* genes and the key flavonoid genes. Four genes (*PfMADS48*, *64*, *77*, and *82*) belong to the *AG* subfamily. Previous studies have reported that the overexpression of *MdJa2*, which belongs to the *AGL11*/*STK* subfamily, in apple trees leads to the inhibition of anthocyanin and proanthocyanidin synthesis (Su et al., 2022). Phylogenetic analysis reveals a close relationship between *PfMADS48, 64, 77*, and *82* and the *AGL11/STK* subfamily. Therefore, these genes (*PfMADS48*/*64*/*77*/*82*) can be considered as potential candidates for further in-depth research.

MADS-box genes, functioning as transcriptional regulators, play a crucial role in ontogeny and signal transduction across various species (Schilling et al., 2018). In recent years, there has been a continuous discovery of a significant number of MIKC-type genes, and their role in regulating abiotic stress has been confirmed (Mou et al., 2009). It has been reported that the majority of *PfMADS* genes, which have known functions in plants, are categorized as Type II (Saedler et al., 2001; Becker and Theissen, 2003). For instance, the expression of *CaMADS* in peppers is influenced by low temperature, salt, and ABA (Wang et al., 2019). The expression of *ZZM7-L* of *AGL12* subfamily is down-regulated in response to NaCl, cold treatment, and mannitol treatment (Zhang et al., 2012). Similar results have been found in other species, such as poplar, soybean, and plum blossom (Leseberg et al., 2006; Shu et al., 2013; Xu et al., 2014). In this study, we focused on six *PfMADS* genes belonging to the MIKC^C^-type in *P. frutescens*. Our objective was to analyze the expression patterns of these genes under cold stress and drought stress (**Figure 11**). The expression of *PfMADS15* and *PfMADS93* was observed to increase significantly under low temperature and drought stress conditions, whereas the expression of *PfbMADS80* was found to decrease significantly under the same conditions. This suggests that three *PfMADS* genes may play distinct roles in the stress response processes of *P. frutescens*, potentially acting as positive and negative regulators. Studies have shown that the kiwifruit *SOC1* gene can activate the *AcSVP2* promoter and identified other desiccation/osmotic stress and dormancy-related transcription factors that may regulate *AcSVP2* expression (Wu et al., 2018). *PfMADS93*(SOC1 subfamily) gene expression significantly increased under drought and cold stress conditions. Therefore, the *PfMADS93* gene can be considered as a potential target gene to test its function in further studies. This would provide evidence for *PfMADS* mediated abiotic stresses in *P. frutescens* and contribute to a new scientific basis for understanding the molecular regulatory mechanism of abiotic stress response and genetic improvement for enhancing resistance in *P. frutescens*.

**Figure 13:**
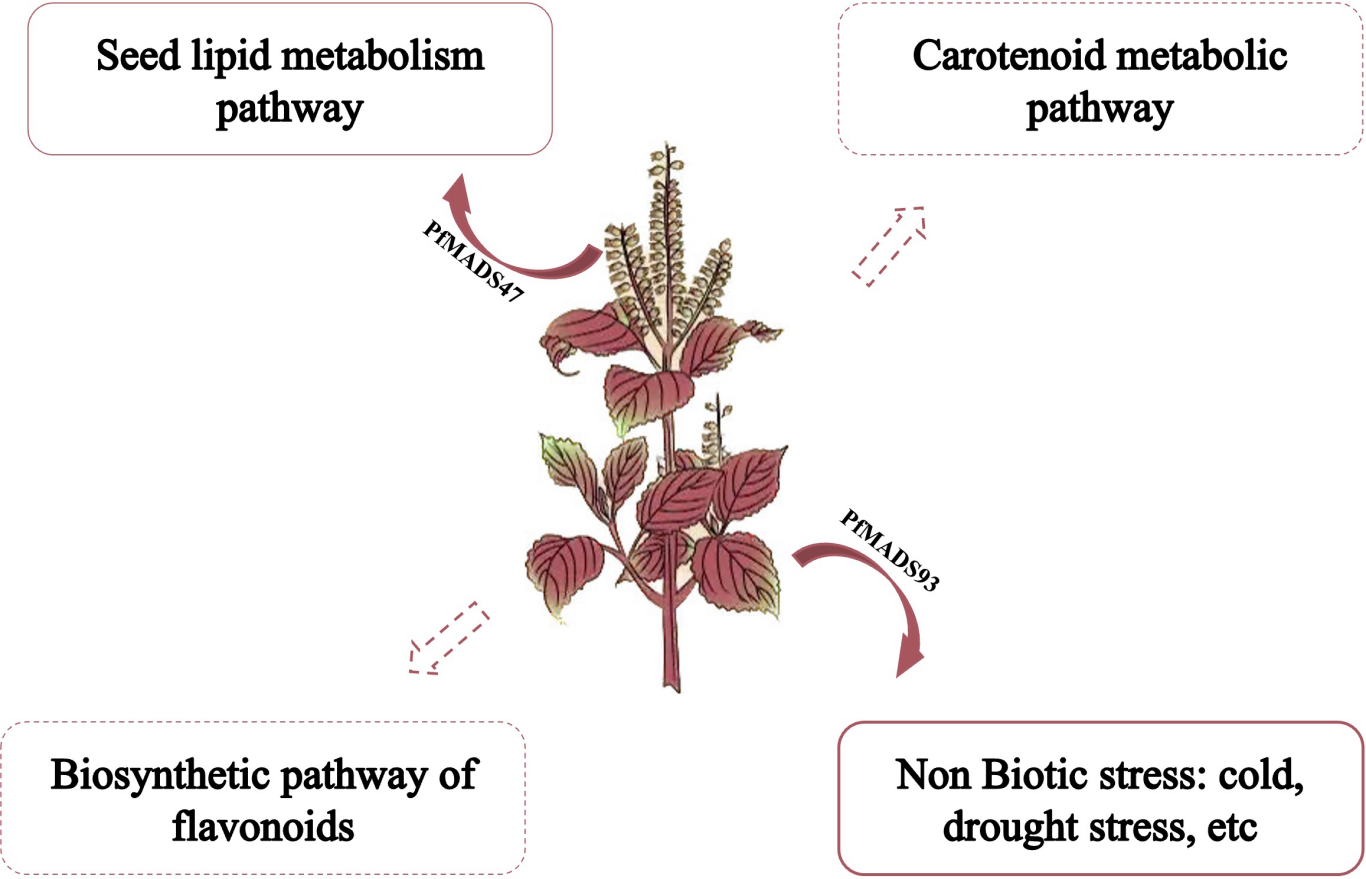
A proposed working model showing the possible roles of *PfMADS* genes in abiotic stress responses and regulating lipid, carotenoid, flavonoids metabolisms in *P. frutescens*.

The most common *P. frutescens* varieties grown in the northwest region of China, such as Shanxi and other places, are primarily cultivated for oil production. However, field cultivation of *P. frutescens* in this region often faces challenges such as low temperature and drought stresses during the seedling and reproductive growth stages (Fang et al., 2022; Zhao et al., 2021). Currently, there is a limited number of studies investigating the molecular mechanism of stress responses, substance synthesis, and metabolism regulation in *P. frutescens*. Therefore, this study established a functional framework for studying the *PfMADS* genes (**Figure 13**). Based on our research, we speculate that *PfMADS* genes may play a role in the accumulation of carotenoids and the metabolic pathway of flavonoids during growth and development. Additionally, it may also be involved in abiotic stress responses. We also screened several candidate genes, including *PfMADS27*, *PfMADS48*, *PfMADS65*, *PfMADS93*, etc. This screening process laid the foundation for further research on the function of the *PfMADS* genes and contributed to the advancement of molecular breeding programs.

## Conclusion

This study has successfully identified 93 *PfMADS* members from the *P. frutescens* genome, marking the first time this has been achieved. The expansion of the *PfMADS* gene family has been primarily driven by segment duplication. Furthermore, it was observed that nearly all *PfMADS* genes have undergone purifying selection throughout their evolutionary history. Over 50% of the *PfMADS* genes exhibit expression in multiple tissues, while certain genes demonstrate tissue-specific expression. These findings suggest variations in the functions of *PfMADS* genes across different tissues in *P. frutescens*. Further studies revealed that several *PfMADS* genes (*PfMADS15*, *22*, *25*, *45*, *80*, *93*) play key roles in response to abiotic stresses, while a group of *PfMADS* genes (*PfMADS6*, *19*, *23*, *27*, *31*, *41*, *42*, *45*, *48*, *61*, *64*, *65*, *77*, *79*, *82*, *91*) may mediate *P. frutescens* seeds development process of lipid metabolism. *PfMADS* genes have been found to be active in all plant tissues that have been studied. These genes form a closely related regulatory network in plants and play various roles in important physiological activities. Our study offers a theoretical foundation for identifying *PfMADS* genes that are involved in flavonoid accumulation, lipid anabolism, and the response to abiotic stresses.

## Data availability statement

The original contributions presented in the study are included in the article/**Supplementary Material**. Further inquiries can be directed to the corresponding authors.

## Author contributions

ML: Writing – original draft. ZD: Methodology, Writing – original draft. ZY: Methodology, Writing – original draft. TL: Software, Writing – original draft. CJ: Writing – review & editing. HC: Supervision, Writing – review & editing. RL: Supervision, Writing – review & editing.

## Supplementary material

Supplementary Figure S1 Identification and screening of transformants of Chlamydomonas reinhardtii. **(A)**. Vector structure diagram of pHR13-PfMADS47. **(B)**. a: Genomic identification of algal transformants; b: RT-PCR analysis of the algal transformants. **(C)** Algal transformants were screened by TAP agar plate containing 10 mg·L−1 hygromycin.

Supplementary Table S1 The list of primer-sets of PfMADS genes for qRT-PCR.

Supplementary Table S2 Detailed information of all identified *P. frutescens* PfMADS proteins.

Supplementary Table S3 The sequence of highly conserved region of the MADS domains.

Supplementary Table S4 Subgenome assignment of tetraploid *P. frutescens*.

Supplementary Table S5 Duplication gene pair of PfMADS.

Supplementary Table S6 Gene ID of MADS-box genes in *Salvia japonica Thunb.*

Supplementary Table S7 Gene ID of MADS-box genes in *Salvia miltiorrhiza.*
