# Supplementary figures and images for: Genome-wide characterization and expression analysis of MADS-box transcription factor gene family in Perilla frutescens
Source: Front Plant Sci. 2024 Jan 8;14:1299902. doi: 10.3389/fpls.2023.1299902 (PMC10801092; doi:10.3389/fpls.2023.1299902)

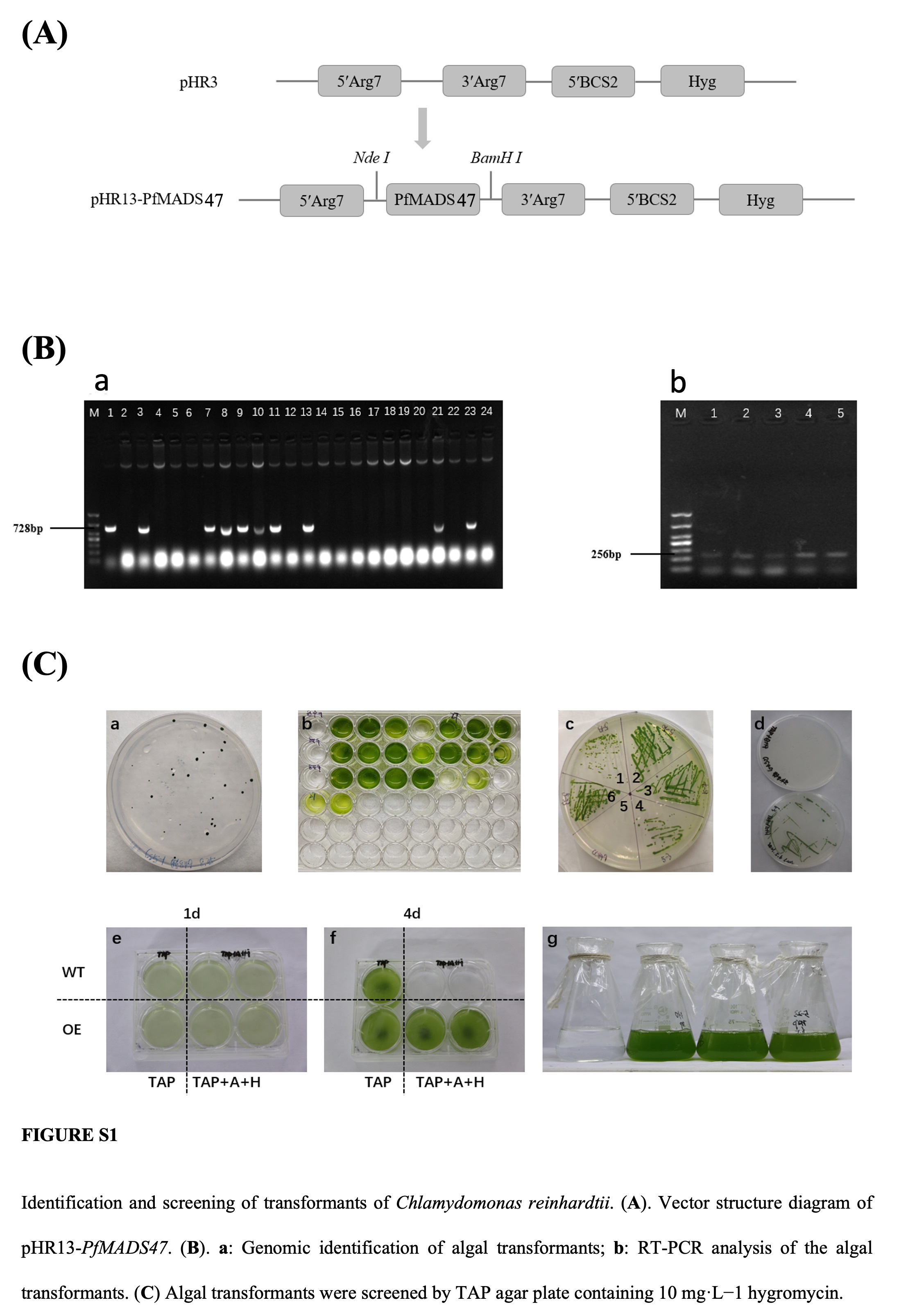

Supplement: Supplementary Figure S1 — Identification and screening of transformants of Chlamydomonas reinhardtii. (A). Vector structure diagram of pHR13-PfMADS47. (B). a: Genomic identification of algal transformants; b: RT-PCR analysis of the algal transformants. (C) Algal transformants were screened by TAP agar plate containing 10 mg·L−1 hygromycin. [file Image_1.jpeg]
