# Supplementary material for: Genome-wide characterization and expression analysis of MADS-box transcription factor gene family in Perilla frutescens
Source: Front Plant Sci. 2024 Jan 8;14:1299902. doi: 10.3389/fpls.2023.1299902 (PMC10801092; doi:10.3389/fpls.2023.1299902)
Supplement: Supplementary Table S1 — The list of primer-sets of PfMADS genes for qRT-PCR. [file DataSheet_1.pdf]

Table S1. The list of primers of *PfMADS* genes for qRT-PCR.

Gene name	Forward primer	Reverse primer
PfMADS15	TGGTGGGCTTGCAGATGAAGATTC	AATGAGTCTCCGAGGTGCTTGAATG
PfMADS22	ATTGATAACTTGACGGCGAGACAGG	GAGGGCAATCTCAGCATCACAGAG
PfMADS25	GGTTGAGCGAAAAGTTGCAGTGTAG	ATTTCTAGTTGCTGGTGGTCCGATG
PfMADS27	GAACAAGGTGAATCAGCAGG	GCAAAACCTCCAATCTAGCC
PfMADS45	CGTCGCCGCCATTGTCATCTC	TCGTTGCAGGATTCCAGTGAAGTTG
PfMADS58	AAAGCTCAAGAACTCGCAAC	TTGAGCTCGAGAGTCAAGTC
PfMADS80	AGGTCCTCCAACGGTATCATAGTCG	GGCTCCTCAAACTCCCTTTCCAC
PfMADS93	GGAAGGTGCAAATGAAGCGAATCG	TCGGCATCGCAAAGAACTGAGAG

Table S2. Detailed information of identified *P. frutescens* PfMADS proteins.

Gene name	Gene ID	Chromosome number	Protein characteristics				CDS length	No of exons	Classification	
			Domain	Length(aa)	MW (kDa)	pl			Group	Subfamily
PfMADS47	KAH6795315.1	chr19	SRF-TF, K-box	251	19.93	9.12	756	6	MIKCC	AG
PfMADS48	KAH6795551.1	chr19	SRF-TF, K-box	253	29.41	9.11	762	7	MIKCC	AG
PfMADS64	KAH6785436.1	chr20	SRF-TF	98	27.59	9.74	246	2	MIKCC	AG
PfMADS77	KAH6773584.1	chr05	SRF-TF, K-box	237	27.39	9.24	596	7	MIKCC	AG
PfMADS81	KAH6763836.1	chr07	SRF-TF, K-box	181	20.72	9.33	546	7	MIKCC	AG
PfMADS82	KAH6764099.1	chr07	SRF-TF, K-box	250	29	9.26	753	7	MIKCC	AG
PfMADS45	KAH6798463.1	chr18	SRF-TF, K-box	198	22.51	8.78	597	7	MIKCC	FLC
PfMADS67	KAH6777493.1	chr04	SRF-TF	198	22.63	7.61	597	7	MIKCC	FLC
PfMADS80	KAH6768970.1	chr06	SRF-TF, K-box	198	22.65	7.59	597	7	MIKCC	FLC
PfMADS11	KAH6813149.1	chr11	SRF-TF, K-box	230	26.71	8.86	693	7	MIKCC	SEP
PfMADS23	KAH6812130.1	chr12	SRF-TF, K-box	230	26.67	8.86	693	7	MIKCC	SEP
PfMADS31	KAH6805456.1	chr15	SRF-TF, K-box	230	26.30	7.64	699	8	MIKCC	SEP
Gene name	Gene ID	Chromosome number	Protein characteristics				CDS length	No of exons	Classification	
			Domain	Length(aa)	MW (kDa)	pl			Group	Subfamily
PfMADS42	KAH6802167.1	chr17	SRF-TF, K-box	234	26.51	7.64	705	8	MIKCC	SEP
PfMADS61	KAH6791248.1	chr02	SRF-TF, K-box	253	29.15	7.71	762	8	MIKCC	SEP
PfMADS79	KAH6774619.1	chr05	SRF-TF, K-box	253	29.13	7.71	762	8	MIKCC	SEP
PfMADS22	KAH6811755.1	chr12	SRF-TF, K-box	168	19.35	10	507	5	MIKCC	SVP
PfMADS29	KAH6808110.1	chr14	SRF-TF, K-box	222	25.21	5.85	669	7	MIKCC	SVP
PfMADS36	KAH6803802.1	chr16	SRF-TF, K-box	151	17.42	9.1	453	5	MIKCC	SVP

PfMADS46	KAH6799430.1	chr18	SRF-TF, K-box	241	27.23	5.94	726	7	MIKCC	SVP
PfMADS58	KAH6789933.1	chr02	SRF-TF, K-box	160	24.53	5.51	663	8	MIKCC	SVP
PfMADS74	KAH6773223.1	chr05	SRF-TF, K-box	220	24.56	9.02	663	8	MIKCC	SVP
PfMADS85	KAH6760116.1	chr08	SRF-TF, K-box	151	17.45	9.27	453	5	MIKCC	SVP
PfMADS27	KAH6807718.1	chr14	SRF-TF, K-box	242	27.85	9.48	729	8	MIKCC	AP1/FUL
PfMADS33	KAH6806448.1	chr15	SRF-TF, K-box	311	35.53	8.28	936	9	MIKCC	AP1/FUL

**Gene name**	**Gene ID**	**Chromosome number**	**Protein characteristics**				**CDS length**	**No of exons**	**Classification**	
			**Domain**	**Length(aa)**	**MW (kDa)**	**pl**			**Group**	**Subfamily**
PfMADS41	KAH6802166.1	chr17	SRF-TF, K-box	156	18.39	9.57	471	4	MIKCC	AP1/FUL
PfMADS62	KAH6791249.1	chr02	SRF-TF, K-box	225	26.03	9.44	678	8	MIKCC	AP1/FUL
PfMADS65	KAH6781686.1	chr03	SRF-TF, K-box	252	29.00	8.39	759	8	MIKCC	AP1/FUL
PfMADS16	KAH6810213.1	chr12	SRF-TF, K-box	210	24.62	5.58	633	7	MIKCC	AP3
PfMADS50	KAH6788359.1	chr02	SRF-TF, K-box	308	36.14	9.38	927	9	MIKCC	AP3
PfMADS63	KAH6791468.1	chr02	SRF-TF, K-box	210	24.68	6.03	633	7	MIKCC	AP3
PfMADS1	KAH6817048.1	chr01	SRF-TF, K-box	173	19.99	9.21	519	6	MIKCC	SOC1
PfMADS4	KAH6817847.1	chr01	SRF-TF, K-box	222	25.18	9.3	669	7	MIKCC	SOC1
PfMADS7	KAH6819461.1	chr01	SRF-TF, K-box	201	23.01	9.73	606	7	MIKCC	SOC1
PfMADS8	KAH6815545.1	chr10	SRF-TF, K-box	210	24.52	9.3	633	7	MIKCC	SOC1
PfMADS25	KAH6809626.1	chr13	SRF-TF, K-box	210	24.48	9.3	633	7	MIKCC	SOC1
PfMADS51	KAH6789521.1	chr02	SRF-TF, K-box	222	25.18	9.3	669	7	MIKCC	SOC1

**Gene name**	**Gene ID**	**Chromosome number**	**Protein characteristics**				**CDS length**	**No of exons**	**Classification**	
			**Domain**	**Length(aa)**	**MW (kDa)**	**pl**			**Group**	**Subfamily**
PfMADS78	KAH6774134.1	chr05	SRF-TF	64	7.36	10.41	195	2	MIKCC	SOC1
PfMADS93	KAH6756237.1	Scaffold01237.1	SRF-TF, K-box	209	19.91	9.47	630	6	MIKCC	SOC1

PfMADS88	KAH6760689.1	chr08	SRF-TF, K-box	213	25.01	6.56	642	7	MIKCC	PI
PfMADS92	KAH6757567.1	Scaffold0908.1	SRF-TF, K-box	213	25.08	6.56	642	7	MIKCC	PI
PfMADS2	KAH6817743.1	chr01	SRF-TF, K-box	249	28.15	8.52	750	8	MIKCC	AGL15
PfMADS5	KAH6818765.1	chr01	SRF-TF, K-box	247	27.91	8.52	744	8	MIKCC	AGL15
PfMADS15	KAH6814369.1	chr11	SRF-TF, K-box	258	29.08	9.11	777	4	MIKCC	AGL15
PfMADS19	KAH6810686.1	chr12	SRF-TF, K-box	258	29.07	9.11	777	8	MIKCC	AGL15
PfMADS34	KAH6803469.1	chr16	SRF-TF, K-box	316	35.74	9.05	951	7	MIKCC	AGL15
PfMADS83	KAH6759781.1	chr08	SRF-TF, K-box	315	35.57	8.54	948	7	MIKCC	AGL15
PfMADS12	KAH6813482.1	chr11	SRF-TF	166	18.83	9.37	501	4	MIKCC	AGL16
PfMADS21	KAH6811746.1	chr12	SRF-TF	166	18.80	9.66	501	4	MIKCC	AGL16
**Gene name**	**Gene ID**	**Chromosome location**	**Protein characteristics**				**CDS length**	**No of exons**	**Classification**	
			**Domain**	**Length(aa)**	**MW (kDa)**	**pl**			**Group**	**Subfamily**
PfMADS54	KAH6789866.1	chr02	SRF-TF, K-box	239	27.59	9.22	720	8	MIKCC	AGL16
PfMADS73	KAH6773197.1	chr05	SRF-TF, K-box	239	27.59	9.22	720	8	MIKCC	AGL16
PfMADS28	KAH6807781.1	chr14	SRF-TF	380	43.53	5.41	1143	8	MIKC*	
PfMADS37	KAH6803856.1	chr16	SRF-TF	317	35.35	5.09	954	10	MIKC*	
PfMADS66	KAH6782262.1	chr03	SRF-TF	326	37.25	5.28	981	11	MIKC*	
PfMADS84	KAH6760115.1	chr08	SRF-TF	74	8.12	4.58	225	3	MIKC*	
PfMADS86	KAH6760187.1	chr08	SRF-TF	312	34.40	4.96	939	8	MIKC*	
PfMADS87	KAH6760188.1	chr08	SRF-TF	317	35.40	4.99	954	10	MIKC*	
PfMADS91	KAH6758900.1	chr09	SRF-TF	326	37.24	5.36	981	11	MIKC*	
PfMADS14	KAH6814089.1	chr11	SRF-TF	172	20.05	7.1	519	1	M 型	Mα
PfMADS17	KAH6810225.1	chr12	SRF-TF	389	41.62	5.73	1170	1	M 型	Mα
PfMADS18	KAH6810226.1	chr12	SRF-TF	326	34.57	6.26	981	1	M 型	Mα
PfMADS30	KAH6805105.1	chr15	SRF-TF	171	18.44	6.34	516	1	M 型	Mα
PfMADS38	KAH6804370.1	chr16	SRF-TF	169	18.47	5.36	510	1	M 型	Mα

Gene name	Gene ID	Chromosome number	Protein characteristics				CDS length	No of exons	Classification	
			Domain	Length(aa)	MW (kDa)	pl			Group	Subfamily
PfMADS39	KAH6804520.1	chr16	SRF-TF	301	33.42	6.37	906	1	M 型	Mα
PfMADS40	KAH6801854.1	chr17	SRF-TF	173	18.72	5.75	522	1	M 型	Mα
PfMADS49	KAH6795798.1	chr19	SRF-TF	293	32.31	5.12	882	1	M 型	Mα
PfMADS55	KAH6789891.1	chr02	SRF-TF	285	31.06	6.09	858	1	M 型	Mα
PfMADS56	KAH6789892.1	chr02	SRF-TF	216	24.14	8.96	651	1	M 型	Mα
PfMADS57	KAH6789893.1	chr02	SRF-TF	325	35.07	8.99	978	1	M 型	Mα
PfMADS59	KAH6790234.1	chr02	SRF-TF	218	24.13	9.3	657	1	M 型	Mα
PfMADS60	KAH6790235.1	chr02	SRF-TF	242	26.86	6.12	729	1	M 型	Mα
PfMADS68	KAH6771865.1	chr05	SRF-TF	308	33.66	5.54	927	1	M 型	Mα
PfMADS69	KAH6772514.1	chr05	SRF-TF	228	25.39	9.13	687	1	M 型	Mα
PfMADS71	KAH6773163.1	chr05	SRF-TF	325	34.99	9.12	978	1	M 型	Mα
PfMADS72	KAH6773164.1	chr05	SRF-TF	216	24.23	8.73	651	1	M 型	Mα
PfMADS75	KAH6773551.1	chr05	SRF-TF	218	24.14	9.58	657	1	M 型	Mα
PfMADS76	KAH6773552.1	chr05	SRF-TF	243	27.03	5.63	732	1	M 型	Mα
PfMADS89	KAH6760752.1	chr08	SRF-TF	297	33.04	6.64	894	1	M 型	Mα
PfMADS90	KAH6760966.1	chr08	SRF-TF	169	18.45	5.48	510	1	M 型	Mα
PfMADS9	KAH6816728.1	chr10	SRF-TF	328	37.84	5.07	987	1	M 型	Mβ
PfMADS10	KAH6816729.1	chr10	SRF-TF	205	23.69	5.57	618	1	M 型	Mβ
PfMADS13	KAH6813716.1	chr11	SRF-TF	302	34.68	9.16	909	1	M 型	Mβ
PfMADS20	KAH6811455.1	chr12	SRF-TF	302	34.75	9.3	909	1	M 型	Mβ
PfMADS24	KAH6808629.1	chr13	SRF-TF	205	23.64	5.57	618	1	M 型	Mβ
PfMADS32	KAH6805761.1	chr15	SRF-TF	269	31.22	7.69	810	1	M 型	Mβ
PfMADS35	KAH6803574.1	chr16	SRF-TF	313	35.62	9.7	942	1	M 型	Mβ
PfMADS43	KAH6802454.1	chr17	SRF-TF	267	30.95	7.7	804	1	M 型	Mβ

PfMADS44	KAH6802457.1	chr17	SRF-TF	264	30.74	7.73	795	1	M 型	Mβ
PfMADS3	KAH6817802.1	chr01	SRF-TF	258	29.38	8.89	777	1	M 型	Mγ
PfMADS6	KAH6818995.1	chr01	SRF-TF	230	26.02	9.23	693	1	M 型	Mγ
PfMADS26	KAH6807528.1	chr14	SRF-TF	159	18.86	9.57	480	1	M 型	Mγ
PfMADS52	KAH6789558.1	chr02	SRF-TF	258	29.39	8.89	777	1	M 型	Mγ
PfMADS53	KAH6789645.1	chr02	SRF-TF	246	27.70	9.15	741	1	M 型	Mγ
PfMADS70	KAH6772890.1	chr05	SRF-TF	246	27.67	9.15	741	1	M 型	Mγ

Table S3. The sequence of highly conserved regions of the MADS domains.

Name	Amino acid sequence
MIKC*	MGRVKLKIKRIENTTNRQVTFSKRKNGLIKKAYELSILCDIDIALLMFSPSDRASLFSGETR
MIKCC	MGRGKIEIKRIENKTSRQVTFSKRRNGLLKKAYELSVLCDAEVALIIFSSRGKLYEF
Mα	KRKIEIKKIEKESXRQVTFSKRRXGLFKKASELCLLCGAEI
Mβ	LSNRLETIFKKASELSTLCDIEVCVIYYG
Mγ	RKKVKLSLIENDRSRKTTFKKRKKGLLKKLHELSTLCGVKACAVIYSP

Table S4. Sub-genome assignment of tetraploid *P. frutescens*.

PF_chr	From	To	Origin	Numbers of PfMADS
Chr01	1	27,751,620	AA	4
Chr01	27,751,621	75,547,337	BB	3
Chr01	75,547,338	76,507,337	AA	0
Chr02	1	75,925,849	BB	14
Chr03	1	73,186,951	AA	2
Chr04	1	68,127,393	AA	1
Chr05	1	66,039,351	AA	12
Chr06	1	63,979,823	BB	1
Chr07	1	62,940,971	AA	2
Chr07	62,940,972	64,111,010	BB	0
Chr08	1	62,644,896	AA	8
Chr09	1	63,810,348	BB	1
Chr10	1	63,122,535	AA	3
**PF_chr**	**From**	**To**	**Origin**	**Numbers of PfMADS**
Chr11	1	2,396,516	AA	2
Chr11	2,396,517	59,720,668	BB	4
Chr12	1	59,655,398	AA	8
Chr13	1	56,513,981	BB	2
Chr14	1	56,187,003	AA	4
Chr15	1	54,298,802	AA	4
Chr16	1	52,707,976	BB	6
Chr17	1	52,085,854	BB	5

Chr18	1	49,030,717	BB	2
Chr19	1	47,713,458	BB	3
Chr20	1	37,302,395	AA	1
Chr20	37,302,396	37,888,901	BB	0

Table S5. Duplication gene pairs of PfMADS.

Paralogous pairs	Ka	Ks	Ka/Ks	Purifying Selection	Duplicate genes type
KAH6817743.1 and KAH6818765.1	0.005190332	0.038224833	0.1357843	YES	WGD/segmental
KAH6813482.1 and KAH6811746.1	2.283391828	1.982513268	1.151766228	NO	WGD/segmental
KAH6814369.1 and KAH6810686.1	0.005027952	0.03502582	0.143549868	YES	WGD/segmental
KAH6811746.1 and KAH6789866.1	0.153055343	1.279542873	0.119617205	YES	WGD/segmental
KAH6811746.1 and KAH6773197.1	0.15456044	1.311506912	0.117849505	YES	WGD/segmental
KAH6805105.1 and KAH6804370.1	0.484476465	1.183639027	0.409310993	YES	WGD/segmental
KAH6804370.1 and KAH6801854.1	0.509703192	1.221713045	0.417203691	YES	WGD/segmental
KAH6804370.1 and KAH6760966.1	0.020690967	0.035615	0.580962167	YES	WGD/segmental
KAH6795551.1 and KAH6785436.1	0.062536207	0.667550653	0.093680092	YES	WGD/segmental
**Paralogous pairs**	**Ka**	**Ks**	**Ka/Ks**	**Purifying Selection**	**Duplicate genes type**
KAH6795551.1 and KAH6773584.1	0.26288359	2.907060294	0.090429356	YES	WGD/segmental
KAH6795551.1 and KAH6764099.1	0.005113656	0.025212458	0.202822595	YES	WGD/segmental
KAH6790234.1 and KAH6773551.1	0.014103841	0.054199648	0.260220156	YES	WGD/segmental
KAH6785436.1 and KAH6764099.1	0.062536207	0.703702229	0.088867427	YES	WGD/segmental
KAH6773584.1 and KAH6764099.1	0.257589669	5.004702522	0.051469527	YES	WGD/segmental
KAH6811755.1 and KAH6773223.1	0.291374092	0.786394461	0.370519003	YES	WGD/segmental
KAH6789866.1 and KAH6773197.1	0.00353983	0.02018059	0.175407638	YES	WGD/segmental
KAH6789933.1 and KAH6773223.1	0.003883504	0.028372177	0.136877187	YES	WGD/segmental
KAH6802167.1 and KAH6774619.1	0.3038591	3.530456557	0.086067933	YES	WGD/segmental
KAH6813149.1 and KAH6812130.1	0.001863355	0.013216201	0.140990213	YES	WGD/segmental
KAH6805456.1 and KAH6802166.1	0.373082097	2.862553606	0.130331916	YES	WGD/segmental

KAH6804520.1 and KAH6760752.1	0.040735159	0.058056051	0.701652253	YES	WGD/segmental
**Paralogous pairs**	**Ka**	**Ks**	**Ka/Ks**	**Purifying Selection**	**Duplicate genes type**
KAH6808110.1 and KAH6803802.1	0.054731027	0.955106325	0.057303596	YES	WGD/segmental
KAH6808110.1 and KAH6799430.1	0.015456995	0.073363267	0.210691208	YES	WGD/segmental
KAH6808110.1 and KAH6760115.1	0.263464299	1.563531999	0.168505857	YES	WGD/segmental
KAH6803802.1 and KAH6799430.1	0.057892229	0.873427692	0.06628165	YES	WGD/segmental
KAH6799430.1 and KAH6760115.1	0.242156442	1.191666933	0.203208158	YES	WGD/segmental
KAH6789891.1 and KAH6773163.1	0.652063724	1.92415998	0.338882282	YES	WGD/segmental
KAH6817048.1 and KAH6774134.1	0.161977515	0.512857533	0.315833355	YES	WGD/segmental
KAH6815545.1 and KAH6809626.1	0.004067807	0.029780587	0.136592558	YES	WGD/segmental
KAH6805105.1 and KAH6801854.1	0.023469972	0.041619554	0.563916966	YES	WGD/segmental
KAH6803802.1 and KAH6760116.1	0.005628544	0.010453131	0.538455349	YES	WGD/segmental
KAH6807718.1 and KAH6791248.1	0.519152949	2.809181098	0.184805796	YES	WGD/segmental
KAH6791248.1 and KAH6781686.1	0.50291451	3.197284231	0.157294276	YES	WGD/segmental
**Paralogous pairs**	**Ka**	**Ks**	**Ka/Ks**	**Purifying Selection**	**Duplicate genes type**
KAH6791248.1 and KAH6774619.1	0.001683502	0.043853826	0.038388951	YES	WGD/segmental
KAH6781686.1 and KAH6774619.1	0.515918921	2.312823398	0.223068878	YES	WGD/segmental
KAH6798463.1 and KAH6777493.1	0.24632405	0.919686862	0.267834695	YES	WGD/segmental
KAH6760689.1 and KAH6757567.1	0.001949952	0.024293622	0.080266021	YES	WGD/segmental
KAH6782262.1 and KAH6758900.1	0.009301415	0.04672589	0.199063404	YES	WGD/segmental
KAH6806448.1 and KAH6791249.1	0.300242781	1.535377202	0.19554985	YES	WGD/segmental
KAH6790235.1 and KAH6773552.1	0.021786706	0.062341165	0.349475437	YES	WGD/segmental
KAH6807718.1 and KAH6806448.1	0.323424082	2.89215617	0.111828014	YES	WGD/segmental
KAH6807718.1 and KAH6781686.1	0.22973507	1.020356883	0.225151684	YES	WGD/segmental
KAH6798463.1 and KAH6768970.1	0.252520832	0.919686862	0.27457262	YES	WGD/segmental

KAH6803856.1 and KAH6760187.1	0.831340726	2.35654126	0.352780042	YES	WGD/segmental
KAH6803469.1 and KAH6759781.1	0.015122519	0.009573325	1.579651728	NO	WGD/segmental

**Paralogous pairs**	**Ka**	**Ks**	**Ka/Ks**	**Purifying Selection**	**Duplicate genes type**
KAH6813716.1 and KAH6811455.1	0.016012253	0.111108492	0.144113674	YES	WGD/segmental
KAH6805761.1 and KAH6802454.1	0.033248687	0.057837978	0.574859096	YES	WGD/segmental
KAH6816728.1 and KAH6808629.1	0.15026122	0.236371345	0.635699815	YES	WGD/segmental
KAH6817802.1 and KAH6789558.1	0.008250908	0.006097595	1.353141507	NO	WGD/segmental
KAH6810213.1 and KAH6791468.1	0.014028465	0.057562486	0.243708464	YES	WGD/segmental
