# Supplementary material for: Genome-wide characterization and expression analysis of MADS-box transcription factor gene family in Perilla frutescens
Source: Front Plant Sci. 2024 Jan 8;14:1299902. doi: 10.3389/fpls.2023.1299902 (PMC10801092; doi:10.3389/fpls.2023.1299902)
Supplement: Supplementary Table S6 — Gene ID of MADS-box genes in Salvia japonica Thunb. [file DataSheet_2.pdf]

Salvia japonica Thunb. MADS-box ID
KAG6432982.1
KAG6436298.1
KAG6421930.1
KAG6424992.1
KAG6382569.1
KAG6416690.1
KAG6419315.1
KAG6407218.1
KAG6416802.1
KAG6419404.1
KAG6406755.1
KAG6409200.1
KAG6409102.1
KAG6430195.1
KAG6411572.1
KAG6417441.1
KAG6406736.1
KAG6432993.1
KAG6410614.1
KAG6411380.1
KAG6400327.1
KAG6402533.1
KAG6385157.1
KAG6404029.1
KAG6412282.1
KAG6383105.1
KAG6401749.1
KAG6426716.1
KAG6401187.1
KAG6429527.1
KAG6384393.1
KAG6425508.1
KAG6436310.1
KAG6399003.1
KAG6413297.1
KAG6384529.1
KAG6410712.1
KAG6396703.1
KAG6394319.1
KAG6427982.1
KAG6431029.1
KAG6388873.1
KAG6427985.1
KAG6390247.1
KAG6412118.1
KAG6431028.1
KAG6433192.1
KAG6386256.1
KAG6385686.1
KAG6412337.1
KAG6386014.1
KAG6386255.1
KAG6387631.1
KAG6387571.1
KAG6387544.1
KAG6385807.1
KAG6412229.1

KAG6386142.1
KAG6397064.1
KAG6427562.1
KAG6436301.1
KAG6432985.1
KAG6397119.1
KAG6421292.1
KAG6429911.1
KAG6433525.1
KAG6422422.1
KAG6408153.1
KAG6408152.1
KAG6405858.1
KAG6429810.1
KAG6435706.1
KAG6400464.1
KAG6405564.1
KAG6429811.1
KAG6426186.1
KAG6410234.1
KAG6412832.1
KAG6390047.1
KAG6412318.1
KAG6392094.1
KAG6393844.1
KAG6416691.1
KAG6434608.1
KAG6437962.1
KAG6397043.1
KAG6386251.1
KAG6426187.1
KAG6399004.1
KAG6405563.1
KAG6392568.1
KAG6414934.1
KAG6432992.1
KAG6392728.1
KAG6385739.1
KAG6387575.1
KAG6409201.1
KAG6406828.1
KAG6420729.1
KAG6419283.1
KAG6416822.1
KAG6387123.1
KAG6387107.1
KAG6437585.1
KAG6427969.1
KAG6431009.1
KAG6426488.1
KAG6408793.1
KAG6397399.1
KAG6399565.1
KAG6421828.1
KAG6382309.1
KAG6425193.1
KAG6385281.1
KAG6387923.1

KAG6409665.1
KAG6389537.1
KAG6418980.1
KAG6405158.1
KAG6431042.1
KAG6435895.1
KAG6389733.1
KAG6391459.1
