# Supplementary material for: Genome-wide characterization and expression analysis of MADS-box transcription factor gene family in Perilla frutescens
Source: Front Plant Sci. 2024 Jan 8;14:1299902. doi: 10.3389/fpls.2023.1299902 (PMC10801092; doi:10.3389/fpls.2023.1299902)
Supplement: Supplementary Table S7 — Gene ID of MADS-box genes in Salvia miltiorrhiza. [file DataSheet_3.pdf]

Salvia miltiorrhiza MADS-box ID
XP_057763603.1
XP_057763604.1
XP_057763605.1
XP_057763606.1
XP_057763607.1
XP_057763608.1
XP_057763609.1
XP_057763610.1
XP_057763611.1
XP_057763612.1
XP_057763613.1
XP_057763614.1
XP_057763615.1
XP_057763616.1
XP_057763617.1
XP_057763620.1
XP_057763747.1
XP_057764223.1
XP_057764224.1
XP_057764225.1
XP_057764226.1
XP_057764227.1
XP_057764233.1
XP_057764242.1
XP_057764252.1
XP_057764253.1
XP_057764254.1
XP_057765952.1
XP_057766037.1
XP_057766039.1
XP_057766054.1
XP_057766055.1
XP_057766147.1
XP_057766148.1
XP_057766346.1
XP_057766347.1
XP_057766348.1
XP_057766349.1
XP_057767486.1
XP_057767487.1
XP_057767488.1
XP_057767489.1
XP_057767490.1
XP_057767491.1
XP_057767493.1
XP_057767494.1
XP_057769824.1
XP_057769825.1
XP_057769826.1
XP_057769827.1
XP_057769828.1
XP_057769829.1
XP_057773372.1
XP_057773386.1
XP_057773387.1
XP_057773388.1
XP_057773389.1

XP_057773390.1
XP_057773392.1
XP_057774444.1
XP_057774445.1
XP_057774446.1
XP_057774447.1
XP_057775680.1
XP_057775681.1
XP_057775682.1
XP_057775688.1
XP_057775689.1
XP_057776988.1
XP_057776989.1
XP_057776990.1
XP_057776991.1
XP_057776992.1
XP_057776993.1
XP_057776994.1
XP_057777485.1
XP_057777486.1
XP_057777487.1
XP_057777488.1
XP_057777489.1
XP_057777490.1
XP_057777492.1
XP_057777496.1
XP_057777519.1
XP_057779450.1
XP_057780209.1
XP_057780218.1
XP_057780282.1
XP_057781447.1
XP_057781457.1
XP_057783760.1
XP_057783873.1
XP_057784312.1
XP_057784706.1
XP_057784707.1
XP_057786076.1
XP_057786913.1
XP_057787211.1
XP_057788058.1
XP_057788221.1
XP_057788222.1
XP_057788223.1
XP_057788224.1
XP_057788225.1
XP_057788226.1
XP_057793675.1
XP_057793676.1
XP_057793694.1
XP_057793880.1
XP_057794379.1
XP_057797658.1
XP_057797659.1
XP_057797943.1
XP_057797983.1
XP_057798504.1

XP_057799193.1
XP_057799397.1
XP_057801257.1
XP_057802816.1
XP_057802968.1
XP_057803749.1
XP_057805016.1
XP_057805017.1
XP_057805154.1
XP_057805155.1
XP_057805156.1
XP_057805691.1
XP_057807158.1
XP_057807458.1
XP_057807574.1
XP_057812383.1
